# Analysis of an Intrinsic Mycovirus Associated With Reduced Virulence of the Human Pathogenic Fungus *Aspergillus fumigatus*

**DOI:** 10.3389/fmicb.2019.03045

**Published:** 2020-01-17

**Authors:** Azusa Takahashi-Nakaguchi, Erika Shishido, Misa Yahara, Syun-ichi Urayama, Kanae Sakai, Hiroji Chibana, Katsuhiko Kamei, Hiromitsu Moriyama, Tohru Gonoi

**Affiliations:** Medical Mycology Research Center, Chiba University, Chiba, Japan

**Keywords:** *Aspergillus fumigatus*, dsRNA, mycovirus, hypovirulence, Chrysoviridae

## Abstract

*Aspergillus fumigatus* is an airborne fungal pathogen that causes severe infections with invasive growth in immunocompromised patients. Several mycoviruses have recently been isolated from *A. fumigatus* strains, but there are presently no reports of mycoviral-mediated reduction or elimination of fungal pathogenicity in vertebrate models. Here, we report the biological features of a novel mycovirus, *A. fumigatus* chrysovirus 41362 (AfuCV41362), isolated from the hypovirulent *A. fumigatus* strain IFM 41362. The AfuCV41362 genome is comprised of four dsRNAs, each of which contains a single ORF (ORF1-4). ORF1 encodes a protein with sequence similarity to RNA-dependent RNA polymerases of viruses in the family Chrysoviridae, while ORF3 encodes a putative capsid protein. Viral RNAs are expressed primarily during the germination stage, and RNA-seq analysis of virus-infected *A. fumigatus* at the germination stage suggested that the virus suppressed expression of several pathogenicity-associated host genes, including hypoxia adaptation and nitric oxide detoxification genes. *In vitro* functional analysis revealed that the virus-infected strain had reduced tolerance to environmental stressors. Virus-infected *A. fumigatus* strain IFM 41362 had reduced virulence *in vivo* compared to the virus-free strain in a mouse infection model. Furthermore, introduction of the mycovirus to a natively virus-free KU *A. fumigatus* strain induced virus-infected phenotypes. To identify mycovirus genes responsible for the reduced virulence of *A. fumigatus*, each viral ORF was ectopically expressed in the virus-free KU strain. Ectopic expression of the individual ORFs only nominally reduced virulence of the host fungus in a mouse infection model. However, we found that ORF3 and ORF4 reduced tolerance to environmental stresses in *in vitro* analysis. Based on these results, we suggest that the AfuCV41362 mycovirus ORF3 and ORF4 reduce fungal virulence by suppressing stress tolerance together with other viral genes, rather than alone.

## Introduction

The filamentous fungus *Aspergillus fumigatus* is the primary cause of aspergillosis, a life-threatening infection in immunosuppressed patients. Novel therapeutic modalities for treatment of aspergillosis are needed to overcome emerging resistance to antifungal drugs.

Mycoviruses selectively infect fungi, are widely distributed in fungal groups, and typically possess RNA genomes. Some dsRNA mycoviruses of phytopathogenic fungi significantly decrease pathogenicity of their fungal hosts, suggesting great potential for control of the corresponding fungal diseases (Osaki et al., 2002; Hillman et al., 2004; Nuss, 2005; Chiba et al., 2009; Urayama et al., 2010; Wu et al., 2012; Jia et al., 2014). However, mycoviruses that reduce the virulence of fungal pathogens of animals and humans are not well-characterized, prohibiting the application of mycoviruses as therapeutic modalities for human fungal disease.

Four *A. fumigatus*-infecting mycoviruses have been isolated, including partitivirus-1 (AfuPV-1), chrysovirus (AfuCV, AthCV1), and tetramycovirus-1 (AfuTmV-1). AfuPV-1 does not cause any obvious symptoms in the fungal host (Bhatti et al., 2011), while AfuCV affects host colony morphology, but not virulence of the fungal host (Jamal et al., 2010). AthCV1 reduces conidia formation (Ejmal et al., 2018). AfuTmV-1 modestly suppresses *A. fumigatus* virulence in an insect model, but does not affect the host animal’s fungal burden (Kanhayuwa et al., 2015).

Several mycoviruses proliferate in their hosts by suppressing RNA silencing, the fungal host’s antiviral defense response (Wang and Metzlaff, 2005; Ding and Voinnet, 2007). However, little is known about other mycoviral factors that reduce the virulence of fungal hosts, in part because reverse genetic systems are available for only a limited number of mycoviruses. In the study of a mycovirus infecting a phytopathogenic fungus, Urayama et al. (2012) overcame this technical limitation by analyzing the effects of heterologous expression of specific mycoviral genes in the budding yeast *Saccharomyces cerevisiae*. This work identified a candidate gene responsible for growth defects in host fungi (Urayama et al., 2012).

In the present study, we isolated a novel dsRNA mycovirus from the *A. fumigatus* strain IFM 41362. We demonstrated that this virus, a member of the Chrysoviridae family that we have designated AfuCV41362, suppressed the virulence of *A. fumigatus* in a mouse infection model. We investigated the mechanism of suppressed fungal virulence by analyzing RNA expression of infected fungi and ectopically expressing individual mycoviral ORFs in *A. fumigatus*, and assessing fungal morphology and virulence *in vivo*.

## Results

### Nucleotide Sequences of dsRNA and Phylogenetic Analysis

**FIGURE 1:**
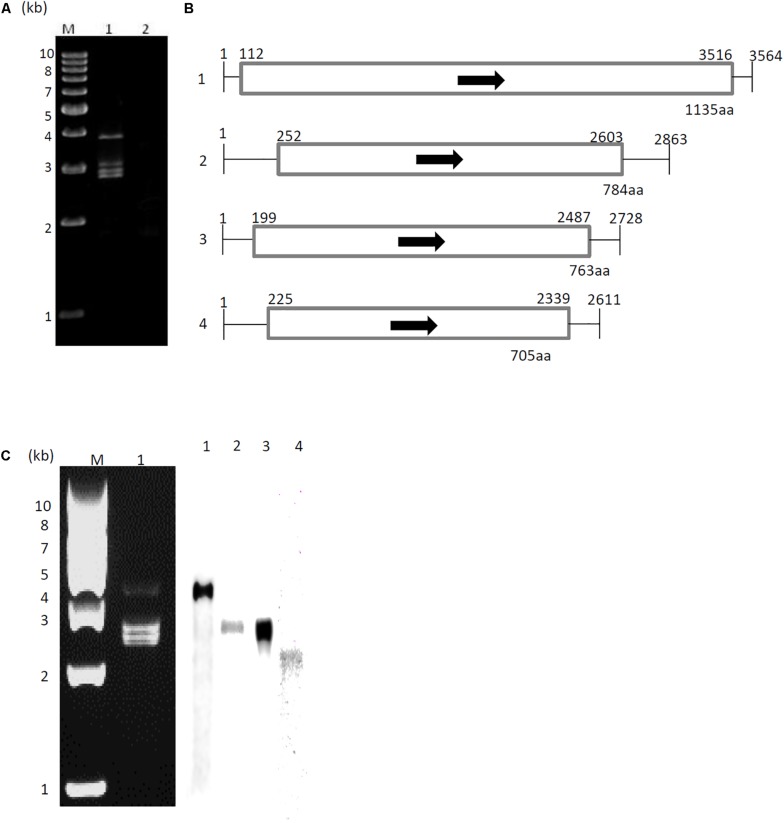
Genome organization of AfuCV41362. **(A)** dsRNA was extracted and electrophoresed on a 5% (wt/vol) polyacrylamide gel. Lane M, molecular weight maker; Lanes 1 and 2, extracts from AfuCV41362-infected and virus-free strains, respectively. **(B)** Schematic drawing of the genomic organization of AfuCV41362. The numbers 1–4 on the left indicate revealed contigs. The horizontal lines and boxes indicate full dsRNA lengths and ORFs, respectively. Arrows indicate ORF sense orientations. Numbers on the lines and boxes indicate nucleotide numbers. Numbers under the boxes indicate amino acid (aa) lengths. **(C) Left:** Agarose gel electrophoresis of dsRNAs purified from *A. fumigatus* strain AfuCV41362. Lane M, molecular weight marker; Lane 1, strain harboring AfuCV41362. **Right:** Northern blot detection of dsRNA fragments using digoxigenin-labeled DNA probes against dsRNA fragments 1–4. dsRNA samples were electrophoresed on four different lanes of a single gel and blotted. The four blotted lanes were separated and hybridized with the probes for each dsRNA. Lanes 1–4 of panel **C** were then reconstituted from the hybridized strips.

dsRNA was extracted from *A. fumigatus* strain IFM 41362 infected with AfuCV41362, and resolved into four bands by polyacrylamide gel electrophoresis (Figure 1A). Following nucleotide sequence analysis, we identified four major contigs, which we designated 1–4 according to decreasing size (Figure 1B). Northern blot analysis using DIG-labeled cDNA probes specific for each of the four dsRNA contigs revealed that the four contigs represented the sequence of the dsRNA bands (Figure 1C).

Sequence analysis demonstrated that dsRNAs 1–4 were 3564, 2863, 2728, and 2611 bp in length, respectively, and that each contig contained a single ORF (Figure 1B). The 5′-untranslated regions (UTRs) of the coding strands of AfuCV41362 dsRNAs 1, 2, 3, and 4 were 111, 251, 198, and 224 nucleotides (nt) long, respectively (Figure 1B and Supplementary Figure S1), and shared 6–7% homology, while the corresponding 3′-UTRs were 48, 260, 241, and 272 nt long, respectively, and shared 11–39% homology (Supplementary Figure S1). In the Chrysoviridae virus family, highly conserved terminal sequences flank both sides of the ORFs in all four segments (Jiang and Ghabrial, 2004). Accordingly, 3′- and 5′-terminal sequences of the four AfuCV41362 dsRNAs had identical nucleic acids.

**FIGURE 2:**
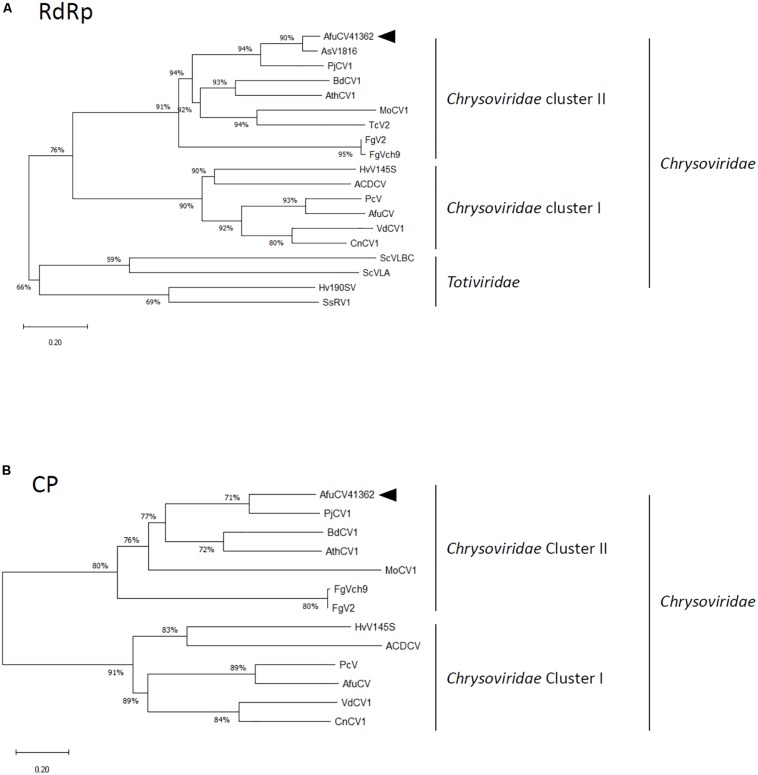
Phylogenetic analysis of **(A)** the putative RNA-dependent RNA polymerase (RdRp) and **(B)** capsid protein (CP) sequences of AfuCV41362 and other selected dsRNA mycoviruses belonging to the families Chrysoviridae and Totiviridae. The phylogenetic tree was generated using the Neighbor Joining (NJ) method. Datasets were subjected to 1000 bootstrap replicates. The scale bars indicate the number of replacements per site. The numbers at the branch points indicate the bootstrap percentage. The full names and sequence information are given in Supplementary Tables S1A,B. The arrowhead in each panel indicates the novel virus described in the present study.

A protein with a molecular weight of 127 kDa was predicted from the 1135-codon ORF carried by dsRNA1 (ORF1; Figure 1B). This amino acid sequence had conserved motifs characteristic of RNA-dependent RNA polymerases (RdRps) of dsRNA viruses infecting simple eukaryotes (Bruenn, 1993). A BLASTP search of the deduced amino acid sequence identified that the ORF1 protein had high sequence homology (90% identity, 95% similarity) to the RdRp encoded by *Aspergillus* mycovirus 1816 (AsV-1816) isolated from *Aspergillus nidulans* (Hammond et al., 2008a). A phylogenetic tree was generated based on the dsRNA1 ORF of AfuCV41362, and the RdRp sequences of 17 selected RNA viruses were compared using the Neighbor Joining (NJ) method (Figure 2A and Supplementary Table S1A). This phylogenetic analysis revealed that AfuCV41362 is closely related to AsV-1816, and sorted with Chrysoviridae cluster II (Ghabrial et al., 2018).

A protein with a molecular mass of 85 kDa was predicted from the 784-codon dsRNA2 ORF (ORF2, Figure 1B). A BLASTP search with the deduced amino acid sequence of ORF2 identified that this protein had sequence homology (45% identity, 60% similarity) to an 80-kDa protein of unknown function encoded by *Penicillium janczewskii* chrysovirus 1 (PjCV1).

A protein with a molecular mass of 82 kDa was predicted from the 763-codon dsRNA3 ORF (ORF3; Figure 1B). A BLASTP search with the deduced amino acid sequence of ORF3 revealed that this protein had sequence homology to an 84-kDa protein encoded by PjCV1 (59% identity, 75% similarity), and to the putative capsid protein of *Botryosphaeria dothidea* chrysovirus (31% identity, 47% similarity) (Wang L. et al., 2014). Phylogenetic analysis of the putative AfuCV41362 capsid protein (ORF3) also suggested that AfuCV41362 belonged to Chrysoviridae cluster II (Figure 2B and Supplementary Table S1B), as was indicated for ORF1. Interestingly, ORF3 of AfuCV41362 exhibited the homology (35% identity) to ORF4 protein of MOCV1 that has been reported to cause apoptosis when forcedly expressed in yeast, but its function is unknown (Urayama et al., 2012).

A protein with a molecular mass of 76 kDa was predicted from the 705-codon dsRNA4 ORF (ORF4; Figure 1B). The deduced amino acid sequence of ORF4 exhibited the strongest homology (58% identity, 72% similarity) to a 70-kDa protein of PjCV1.

### Virus Particles

**FIGURE 3:**
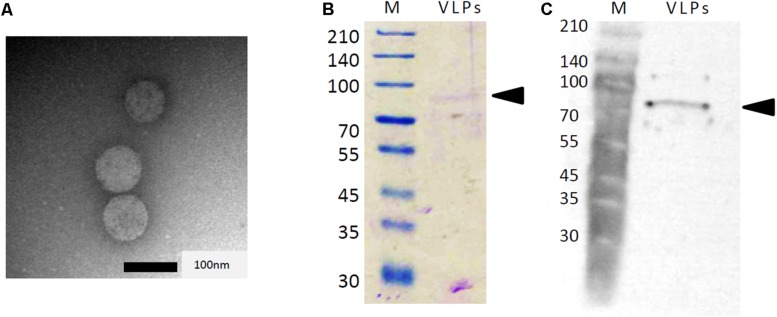
Analysis of AfuCV41362 particles isolated from *A. fumigatus* strain IMF 41362. **(A)** Transmission electron microscope (TEM) photograph negative staining of AfuCV41362 virus particles. **(B)** SDS-PAGE of proteins purified from AfuCV41362. Lane M, molecular weight markers. The gel was stained with Coomassie Brilliant Blue R-250. VLPs, virus-like particles. Arrowheads are indicative of ORF3 band positions. **(C)** Western blot detection of a band by anti-virus ORF3 protein antibody raised against a synthetic peptide for a partial ORF3 sequence.

To determine whether the dsRNAs associated with strain AfuCV41362 were encapsulated, the strain was evaluated for production of virus-like particles (VLPs). As expected, VLPs were detected through centrifugation and gel chromatography, as described in the section “Materials and Methods.” Examination of these particles by transmission electron microscopy (TEM) revealed the presence of isometric VLPs with a diameter of approximately 70 nm (Figure 3A). Agarose gel electrophoresis of the dsRNA extracted from these VLPs revealed that dsRNAs 1–4 were concentrated in the VLPs relative to host DNA and rRNA. SDS–PAGE analysis of the proteins in the VLP-containing fraction revealed the presence of a single major band with an estimated molecular mass of 82 kDa, consistent with the deduced molecular mass of the protein encoded by ORF3 (Figure 3B). Immunoblot analysis using an antibody raised against a partial amino acid sequence of the predicted ORF3 protein (see the section “Materials and Methods”) suggested that ORF3 encoded the major protein of the VLPs (Figure 3C).

### Expression Pattern of AfuCV41362 Genes

**FIGURE 4:**
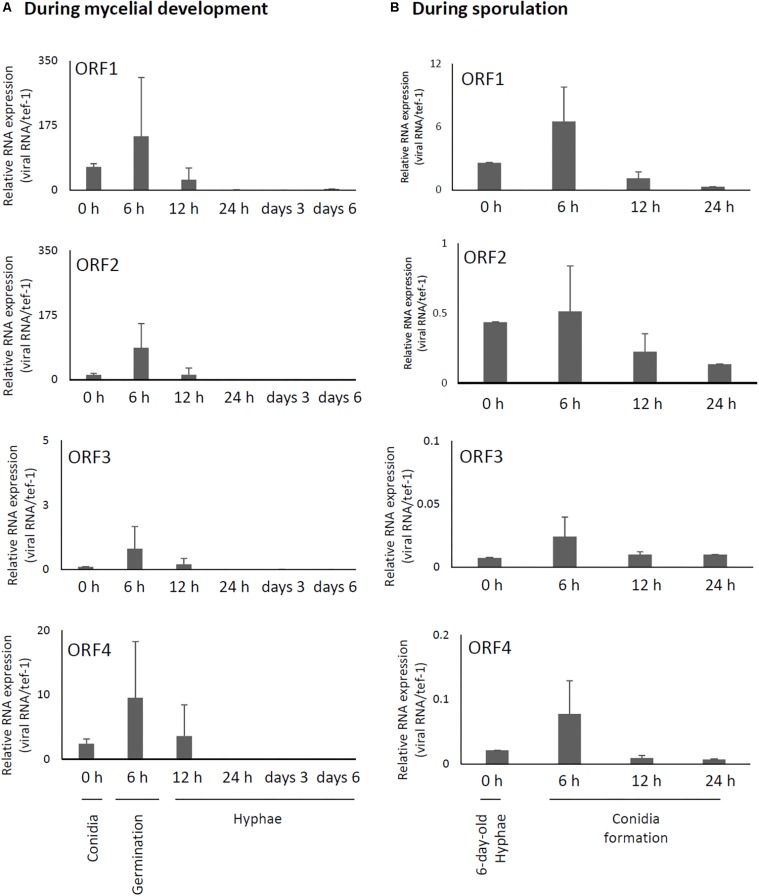
Changes in viral ORF transcript levels at different stages of growth in the AfuCV41362-infected strain. **(A)** RNA levels were analyzed by real-time PCR during the fungal life-cycle, with time points after expose to liquid medium as follows: 0 h (conidia), 6 h (germination), and later stages of mycelial development (12 h, 24 h, and days 3 and 6). **(B)** RNA levels were analyzed by real-time PCR during sporulation, with time points as follows: 0 (day 6, mycelia), 6 (vesicle formation), 12 (phialide formation), and 24 h (conidial formation). Data values were normalized to that of the internal control *tef-1*, and presented as mean ± SD of three independent experiments.

Relative expression levels of AfuCV41362 genes in *A. fumigatus* were examined using real-time quantitative reverse transcriptase-PCR (RT-PCR). These findings suggested that mycoviral RNA levels changed during the progression of fungal development. For all four AfuCV41362 genes (1–4), transcript levels were highest at 6 h after incubation in liquid medium, a time point corresponding to the germination stage of mycelial development (Figure 4A). Transcript levels progressively decreased thereafter during fungal development. On the other hand, during sporulation (Figure 4B), transcript levels of the four ORFs were the highest at a time point corresponding to the vesicle formation stage (6 h). Transcript levels subsequently decreased toward the late conidial maturation stage (24 h). Among the four genes, ORF1 was most highly expressed, with transcripts present at lower levels for ORFs 2, 4, and 3 (in that order) during mycelial development (Figure 4).

### Effect of AfuCV41362 on Fungal Colony Morphology and Mycelial Growth

To investigate whether the mycovirus affected growth and virulence of *A. fumigatus*, we eliminated the mycovirus from the host using a single-spore isolation method. Elimination of the dsRNAs from fungal hyphae of the cured strain was confirmed by dsRNA extraction, agarose gel electrophoresis, and ethidium bromide staining (Supplementary Figure S2). These data suggested that the nominally cured strain was indeed free of AfuCV41362. Therefore, we used this isolate as a virus-free strain in further analyses.

**FIGURE 5:**
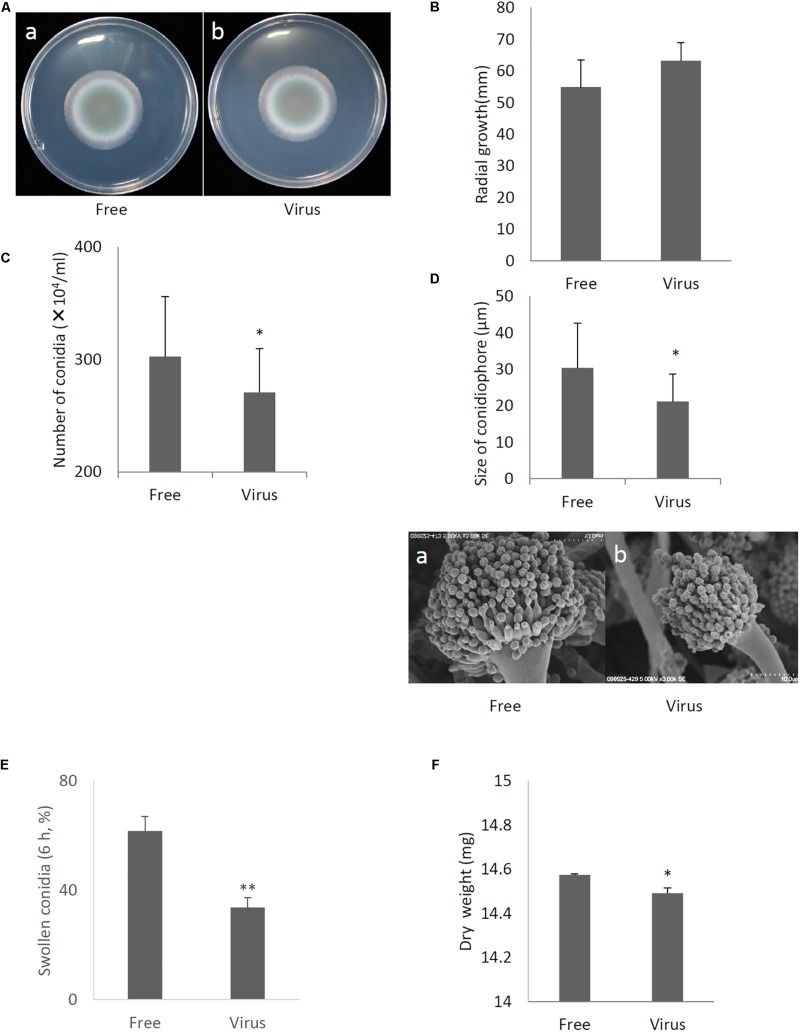
Comparisons of colony morphology and conidial formation of virus-free (Free) and virus-infected (Virus) strains. **(A)** Colony morphology of AfuCV41362 virus-free **(a)** and virus-infected **(b)** *A. fumigatus* strains (cultured for 2 days). **(B)** Radial growth of colonies formed by virus-free and AfuCV41362 virus-infected *A. fumigatus* strains. **(C)** Numbers of conidia formed by the strains at 24 h after starting point inoculation. **(D) Top:** Sizes of conidiophores formed by the virus-free and virus-infected strains. Data are presented as mean + SD of three independent experiments. ^∗^*P* < 0.05, by two-tailed Student’s *t*-test. The data pair of panel **B** was not statistically significant. **Bottom:** SEM images of the virus-free **(a)** and virus-infected **(b)** strains. Calibration, 10 μm. **(E)** Comparisons of conidia swelling of virus-free (Free) and virus-infected (Virus) strains. Percentages of swelling (6 h) conidia formed by the virus-free and virus-infected strains. Data are presented as mean + SD of three independent experiments. ^∗∗^*P* < 0.01, by two-tailed Student’s *t*-test. **(F)** Mycelial growth. Growth was quantified by measuring dry weights of mycelia at 24 h after starting incubation of conidia.

Compared to the original virus-infected *A. fumigatus* IFM 41362 strain, the virus-free strain showed no apparent changes in colony morphology or colony size (Figures 5A,B). However, we observed a 30% reduction in conidial number in the virus-infected strain compared to the virus-free strain (Figure 5C, *P* < 0.05). The reduction of conidial number reflected significant decreases in conidiophore size (Figure 5D, *P* < 0.05). Furthermore, AfuCV41362 also appeared to affect conidial germination, as AfuCV41362 infection resulted in a significant reduction in the number of swollen conidia at 6 h post-inoculation (Figure 5E). The dry weight of mycelium formed in liquid culture at 24 h after starting also was significantly reduced in the virus-infected strain compared to the virus-free strain (Figure 5F, *P* < 0.05).

### Influence of AfuCV41362 on *A. fumigatus* Gene Expression

**FIGURE 6:**
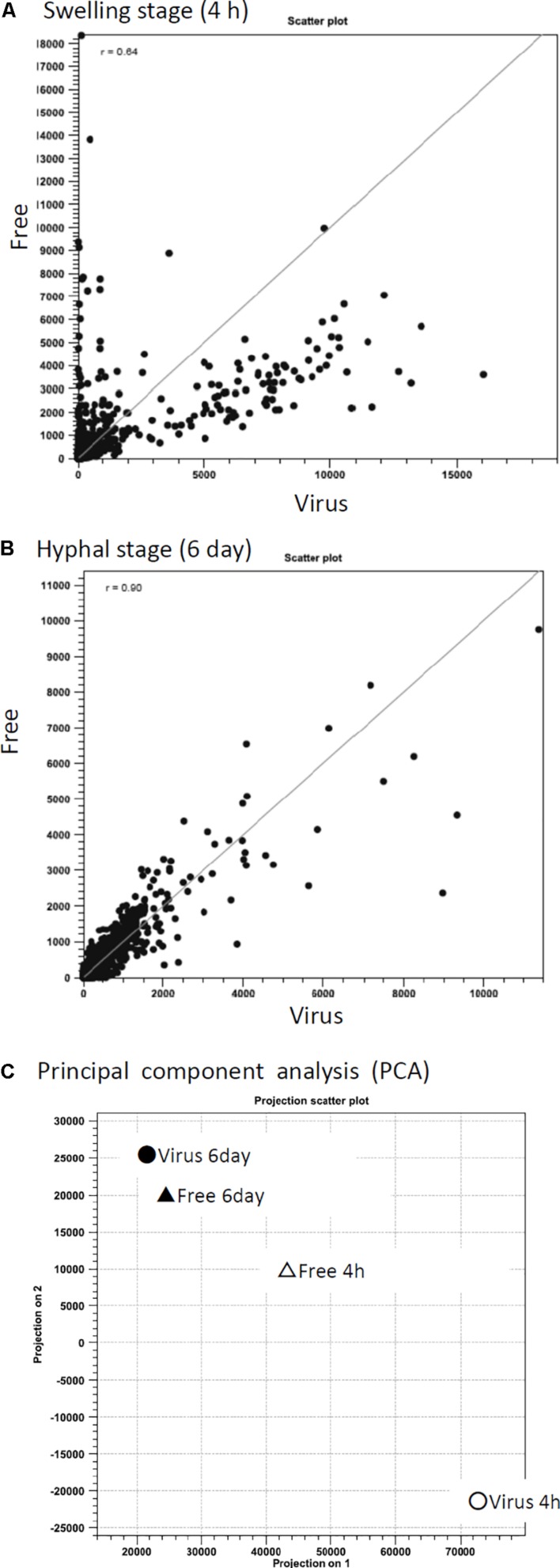
Correlation of differential gene expression between virus-infected (Virus) and virus-free (Free) strains analyzed by RNA-seq. **(A,B)** Scatter plots of transcript levels (RPKMs) at the swelling stage (**A:** 4 h) and the hyphal stage (**B:** day 6). **(C)** Transition of gene expression patterns in the virus-infected (circle symbols) and virus-free (triangle) strains during swelling stage (4 h, white) and hyphal stage (day 6, black). The pattern was examined through principal component analysis (PCA).

To better characterize how *A. fumigatus* responded to AfuCV41362 infection, we performed RNA-seq analyses to compare gene expression between virus-infected and virus-free strains at two distinct time points: just before the start of swelling (4 h after the start of incubation in PDB medium, a stage referred to here as the 4-h swelling stage) and at the hyphal stage (day 6). At the 4-h swelling stage, the pattern of host fungal gene expression dramatically differed between the infected and cured strains (Figures 6A,C).

Gene ontology-like functional catalog (FunCat) analysis was also performed using a web-based application for fungi, FungiFun (Priebe et al., 2011). Genes that changed more than twofold in infected versus non-infected fungi were analyzed. At the 4-h swelling stage, the largest category of down-regulated genes in virus-infected *A. fumigatus* was “C-compound and carbohydrate metabolism” (178 of 1522 down-regulated genes), followed by “secondary metabolism” (162 genes) and “lipid, fatty acid, and isoprenoid metabolism” (127 genes) (Supplementary Figure S3A). At the 4-h swelling stage, the most numerous up-regulated genes were categorized to “protein binding” (451 of 1770 up-regulated genes) followed by “RNA-binding” (117 genes) and “ribosomal proteins” (108 genes) (Supplementary Figure S3B). On day 6 in hyphal stage, the most numerous down-regulated genes were categorized to “lipid, fatty acid, and isoprenoid metabolism” (89 of 1261 down-regulated genes), followed by “regulation by binding/dissociation” (22 genes) (Supplementary Figure S3C). On day 6 in the hyphal stage, the most numerous up-regulated genes were categorized to “protein binding” (206 of 787 up-regulated genes), followed by “transcriptional control” (82 genes) (Supplementary Figure S3D).

Among all of the genes assessed, 13 genes were down-regulated >100-fold (ranging from 101- to 452-fold), 18 genes were down-regulated 50- to 100-fold, and 170 genes were down-regulated 10- to 50-fold in the virus-infected strain compared to the virus-free strain at the 4-h swelling stage (Supplementary Table S2A). In contrast, up-regulated genes in the virus-infected strain at the 4-h swelling stage exhibited smaller (<30-fold) changes compared to the cured strain (Supplementary Table S2B). For gene expression assessed on day 6 after beginning incubation, smaller (<30-fold) changes were detected for both the up- and down-regulated genes between virus-infected and cured strains (Figures 6B,C and Supplementary Tables S2C,D). A total of 228 genes were down-regulated and 202 genes were up-regulated at both time points in the mycovirus-infected strain compared to the cured strain (Supplementary Tables S2E–G).

Among markedly down-regulated genes at 4 h, several transcripts related to carbohydrates, amino acids, and secondary metabolite synthesis were detected (AciA/Fdh; AFUA_6G04920, alcohol dehydrogenase; AFUA_5G06240, PdcA; AFUA_3G11070, indoleamine 2,3-dioxygenase family protein; AFUA_7G02010, isochorismatase family hydrolase; AFUA_6G12220, copper amine oxidase; AFUA_3G14590, acetyl-CoA-acetyltransferase; AFUA_6G14200). Several transcripts related to energy metabolism were also detected (FAD-dependent oxidoreductase; AFUA_7G05070, 3-hydroxyacyl-CoA dehydrogenase; AFUA_5G10070, 6-phosphogluconate dehydrogenase; AFUA_6G08730, flavohemoprotein; AFUA_4G03410, NiiA;AFUA_1G12840). A subset of additional transcripts related to membrane transport was identified (RTA1 domain protein; AFUA_5G05640, MFS multidrug transporter; AFUA_4G01140, MFS monosaccharide transporter; AFUA_5G01160) (Supplementary Table S2A). Among the genes that were down-regulated in virus-infected *A. fumigatus*, the most drastic change was observed in the transcript encoding NAD-dependent formate dehydrogenase AciA/Fdh (452.8-fold reduction), and the second-most strongly down-regulated transcript was that encoding indoleamine 2,3-dioxygenase (IDO) (441.6-fold reduction; Supplementary Table S2A). In the section “Discussion,” we evaluate possible functions of these genes.

Interestingly, expression levels of genes related to RNA silencing (Dicer; AFUA_5G11790 and AFUA_4G02930, and Argonaute; AFUA_3G11010) did not change significantly between infected and cured strains at the time points observed (Supplementary Table S2H).

### Effect of AfuCV41362 on Stress Tolerance of Host Fungi

**FIGURE 7:**
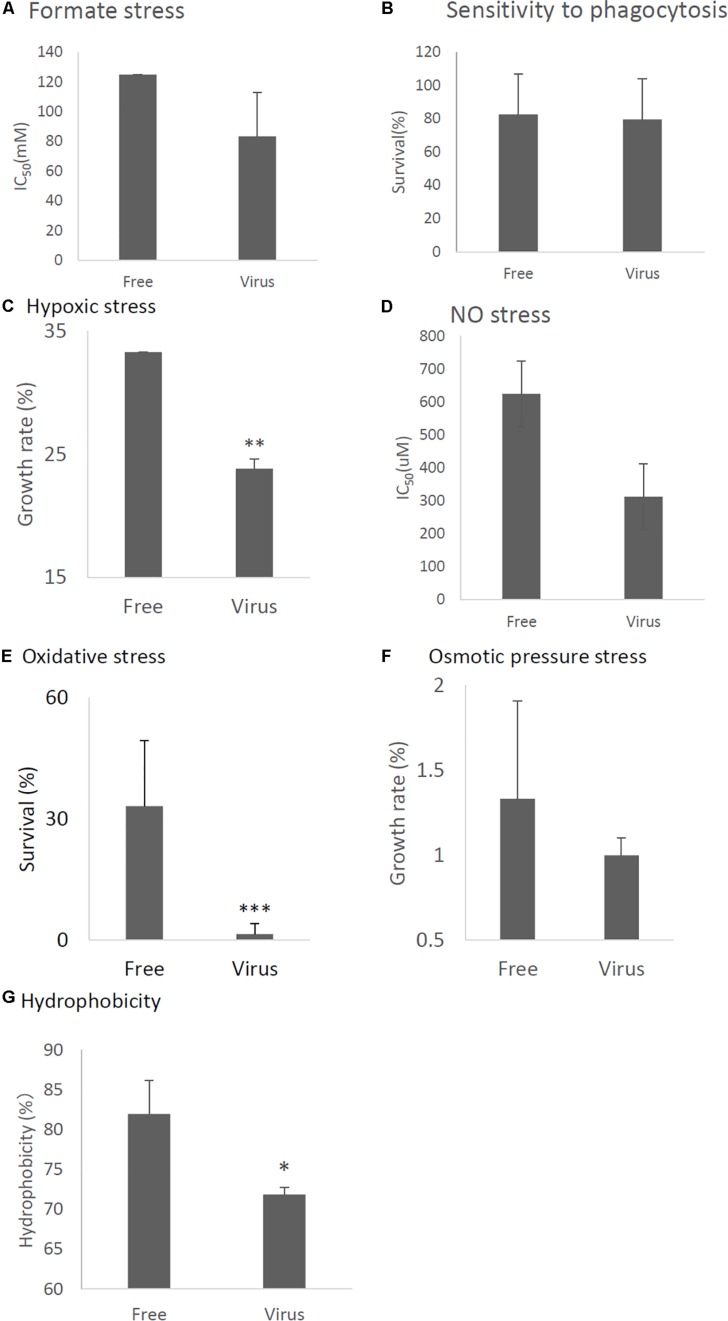
Comparisons of tolerance to stresses of virus-free (Free) and virus-infected (Virus) strains. **(A)** IC_50_ of the strains under formate stress conditions (RPMI 1640 medium). **(B)** Sensitivity of the *A. fumigatus* strains to phagocytosis by J774A.1, a murine macrophage cell line. Techniques are described in the section “Materials and Methods.” **(C)** Effect of hypoxic stress (conidial growth under anaerobic culture conditions). Germination and growth rate are expressed as percentages of growth in the absence of stress. Experimental methods are detailed in the section “Materials and Methods.” **(D)** IC_50_ of the strains under NO stress conditions (NOC-18 in RPMI1640 medium). **(E)** Germination rates of the virus-free and virus-infected strains under oxidative stress (germination following 24-h exposure to 32.6 nM H_2_O_2_). **(F)** Growth of the virus-free and virus-infected *A. fumigatus* strains under osmotic stress conditions (conidial growth in the presence of 0.8 M NaCl). **(G)** Hydrophobicity. Data are presented as mean + SD of three independent experiments. ^∗^*P* < 0.05, ^∗∗^*P* < 0.01, ^∗∗∗^*P* < 0.001, by two-tailed Student’s *t*-test.

In order to examine phenotypic effects of the stress tolerance-related genes down-regulated by viral infection, we compared stress tolerance of virus-infected and virus-free strains. We found AfuCV41362 infection affected fungal response to formate stress (Figure 7A), but did not affect sensitivity to phagocytosis by mouse macrophage J774A.1 cells (Figure 7B). Tolerance to hypoxic stress was significantly reduced in the infected strain (Figure 7C), and growth of the virus-infected strain was also significantly impaired under nitric oxide (NO) and oxidative stresses (Figures 7D,E). Osmotic stresses nominally but non-significantly affected host radial growth in the infected strain (Figure 7F). Hydrophobicity of conidia were reduced in the infected strain (Figure 7G).

### AfuCV41362-Infected *A. fumigatus* Exhibited Hypovirulence in Mice

**FIGURE 8:**
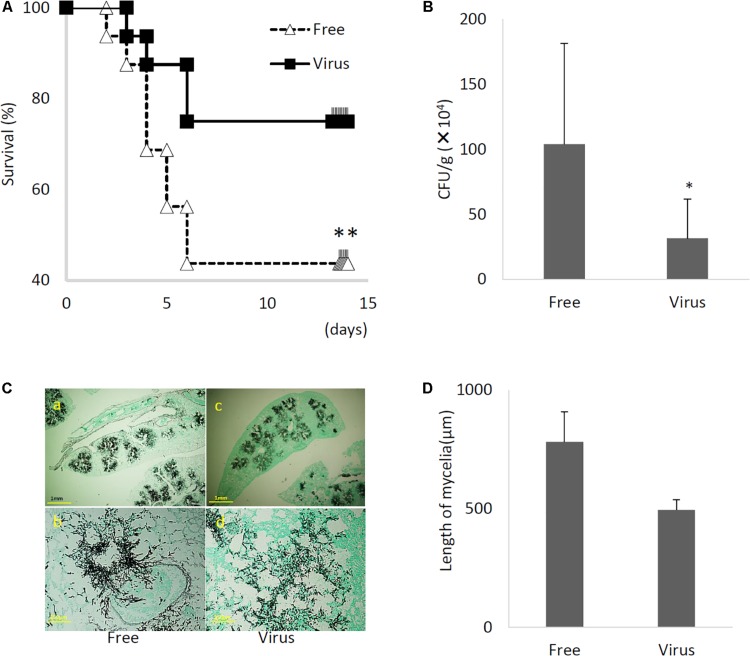
Comparisons of murine virulence of virus-free (Free) and virus-infected (Virus) strains. **(A)** Survival rates of mice infected with the AfuCV41362 virus-infected and virus-free strains. ^∗∗^*P* < 0.05 by Kaplan–Meier log tank test. **(B)** Fungal growth from the lungs of infected mice. Fungal burdens were estimated as CFUs per gram of lung. Five mice per experiments and repeated three times. **(C)** Lung histology of mice at 3 days after infection (Gomori’s methenamine silver-staining-Grocott’s variation). **(a,b)** virus-free strains; **(c,d)** virus-infected strains. **(D)** Quantified mycelial growth in the lungs of mice infected with the virus-free and virus-infected fungal strains. The tissues were dissected for observation on day 3 after infection. For graphs **B** and **D**, data are presented as mean + SD of three independent experiments. ^∗^*P* < 0.05, by two-tailed Student’s *t*-test.

Immunosuppressed mice were infected with virus-infected and virus-free *A. fumigatus* strains, and animal survival was monitored. The virulence of the virus-infected strain was significantly reduced in this infection model (Figure 8A, *P* < 0.01). Furthermore, the lung fungal burden 3 days post-infection was significantly lower in mice infected with the virus-infected fungal strain than in mice infected with virus-free strain (Figure 8B, *P* < 0.01). Histological analyses examined at 3 days post-infection revealed apparent differences between tissues infected with the virus-infected strain and tissues infected with the virus-free strain (Figure 8C). Specifically, while the virus-free strain appeared to penetrate to the lung epithelium and achieve hyphal production in the murine airway, the virus-infected strain remained within the epithelial boundary of the airspace (Figures 8C,D). Taken together, these data demonstrated that the virus-infected strain had a markedly less-invasive phenotype in immunosuppressed murine hosts.

### Infection of Natively Virus-Free KU Strain With AfuCV41362 Virus

**FIGURE 9:**
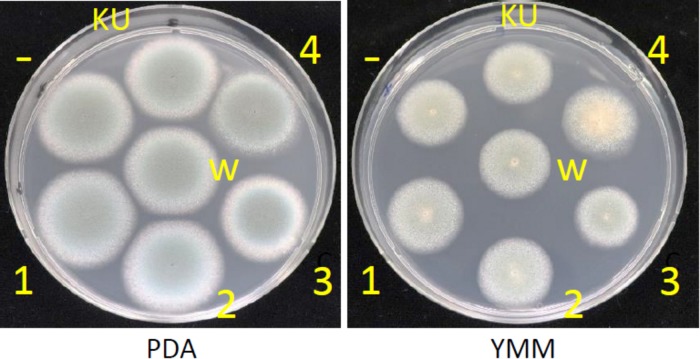
Colony morphologies of the whole-virus infected KU strain, AfuCV41362 ORF-expressing KU strains and the native virus-free strain. 37°C, 24 h. KU, the virus-free KU strain. w, the whole-virus infected KU strain, 1–4, KU strains with forced expression of ORFs (introduced by transformation). -, KU strain carrying an empty vector.

**FIGURE 10:**
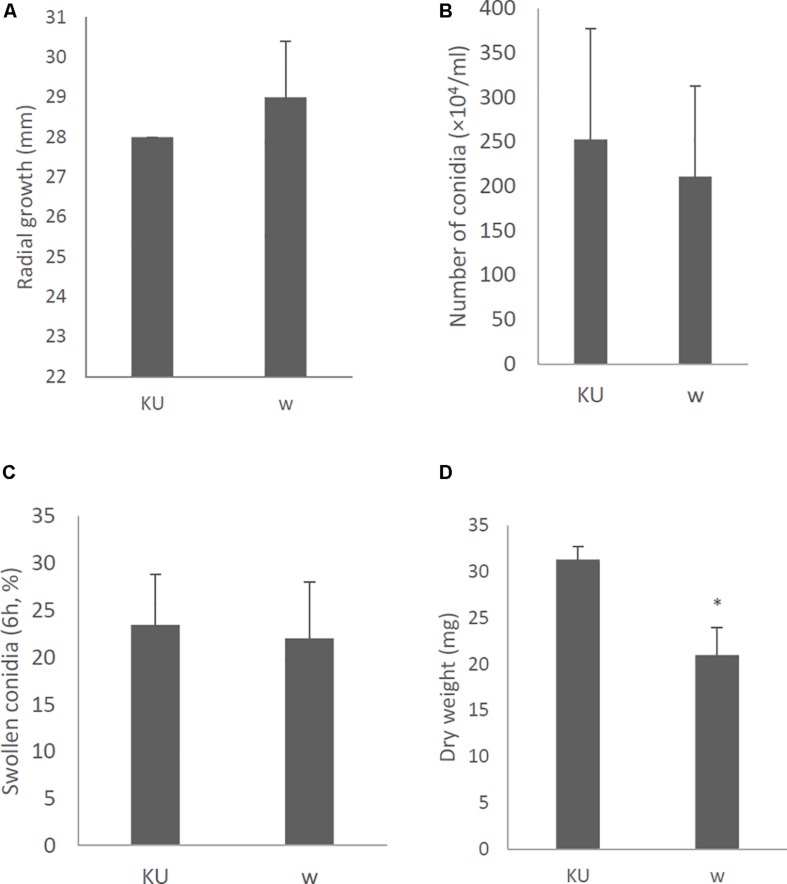
Morphological comparisons of the whole-virus infected KU strain and the native virus-free KU. **(A)** Radial growth of colonies. **(B)** The number of conidia formed by the strains at 24 h after starting point inoculation. **(C)** Percentages of swelling (6 h, D conidia of the strains). **(D)** Mycelial growth. Growth was quantified by measuring dry weights of mycelia at 24 h after starting incubation of conidia. Data are presented as mean ± SD of three independent experiments. ^∗^*P* < 0.05, by two-tailed Student’s *t*-test.

Effects of AfuCV41362 mycovirus infection on *A. fumigatus* were further studied by introducing the mycovirus into the originally virus-free KU strain (AfS35, FGSC A1159, akuA:loxP) via the protoplast fusion method. Infection of the virus was confirmed by agarose gel electrophoresis of dsRNAs extracted from the host (Supplementary Figure S4). The virus-infected KU strain showed no apparent changes in colony morphology (Figure 9), colony size, or conidial number (Figures 10A,B, respectively) compared with the virus-free KU strain. Conidial germination was not affected by virus infection of the KU strain (Figure 10C), while mycelial growth was decreased by infection (Figure 10D).

**FIGURE 11:**
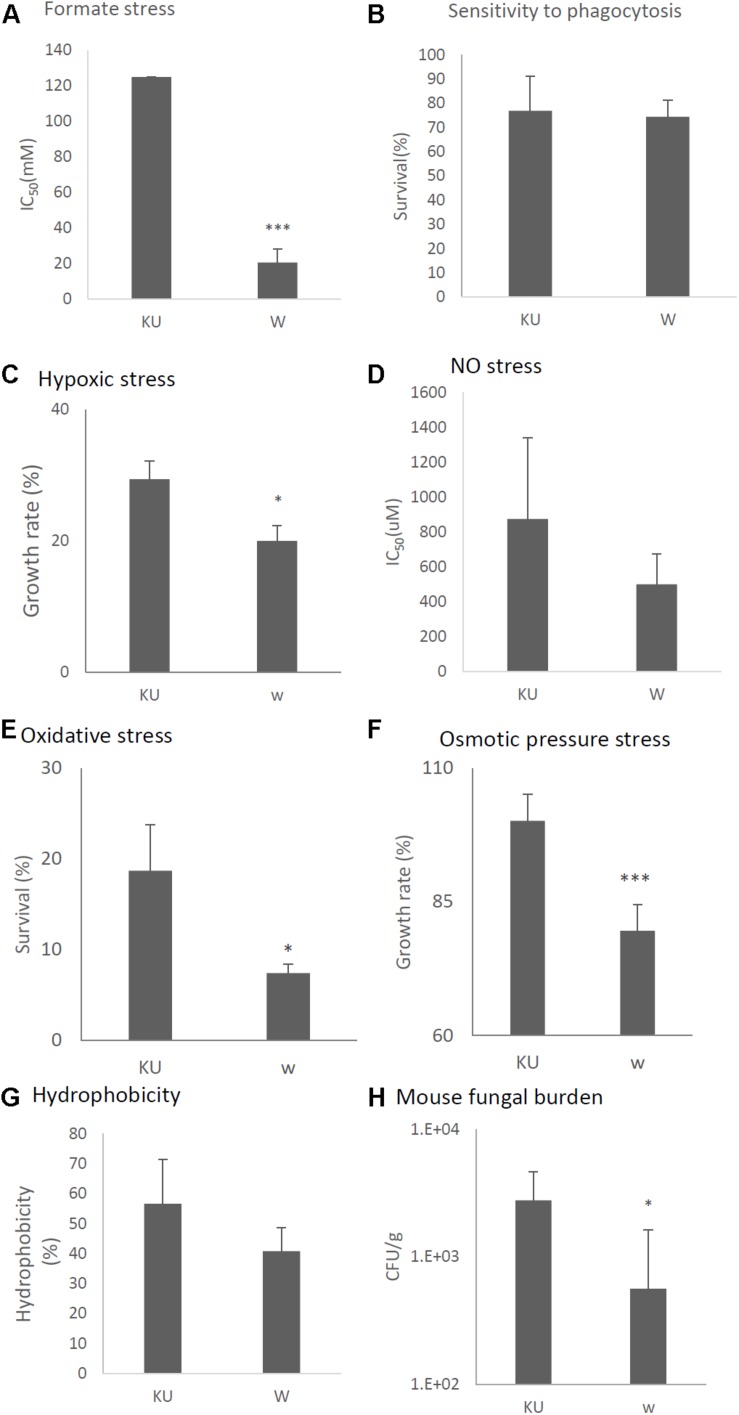
Comparisons of tolerance to stresses, response of mammalian cells, and virulence of the virus-infected KU strain and KU strain. KU, the virus-free KU strain. w, the virus-infected KU strain. The labels are common from **A** to **G**. **(A)** IC_50_ of the strains under formate stress conditions (RPMI 1640 medium). **(B)** Sensitivity of the *A. fumigatus* strains to phagocytosis by A.1, a murine macrophage cell line. Techniques are described in the section “Materials and Methods.” **(C)** Growth of the fungal strains under hypoxic stress conditions. **(D)** IC_50_ of the strains under NO stress conditions (NOC-18 in RPMI 1640 medium). **(E)** Effect of hydrogen peroxide on the growth of the fungal strains. **(F)** Growth of the fungal strains under osmotic stress conditions. **(G)** Hydrophobicity. **(H)** The number of colonies per gram of lung tissue isolated from mice at 72 h after fungal infection. Data are presented as mean ± SD using four mice per group. ^∗^*P* < 0.05. For panels **(B–G)**, data are presented as mean ± SD of three independent experiments. ^∗^*P* < 0.05, ^∗∗∗^*P* < 0.001 by two-tailed Student’s *t*-test comparison of the virus-infected (w) strains to the KU strain.

Tolerance to formate, hypoxic, NO, oxidative, osmotic stresses, and hydrophobicity was also decreased in the virus-infected strain (Figures 11A,C–G, respectively). Viral infection of the KU strain did not affect sensitivity to phagocytosis, as was the case for the natural virus-infected strain (Figure 11B, also see Figure 7B). Virulence of the virus-infected KU was then tested in immunosuppressed ICR mice. Three days post-infection, lung fungal burden was significantly decreased in mice infected with the virus-infected KU strain relative to mice infected with the virus-free KU strain (Figure 11H).

### Expression of Individual AfuCV41362 ORFs Induced Changes in Fungal Morphology and Virulence

We attempted to identify which AfuCV41362 gene products caused hypovirulence using an *A. fumigatus* plasmid expression system. Specifically, the pCB1004 plasmid vector was used to induce ectopic expression of each AfuCV41362 ORF in the KU virus-free strain.

**FIGURE 12:**
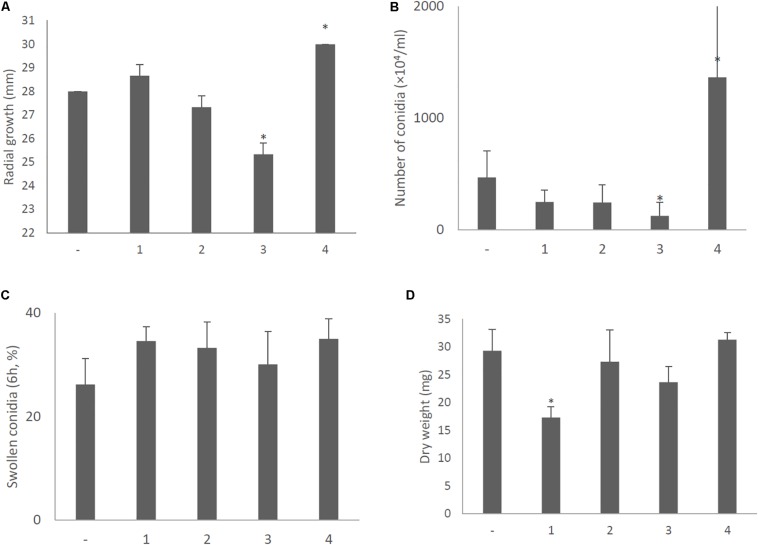
Morphological comparisons of the AfuCV41362 ORF-expressing KU strains. **(A)** Radial growth of colonies by ORF-expressing KU strains. **(B)** The number of conidia formed by the strains at 24 h after starting point inoculation. **(C)** Percentages of swelling (6 h, D conidia of the strains). **(D)** Mycelial growth. Growth was quantified by measuring dry weights of mycelia at 24 h after starting incubation of conidia. Data are presented as mean ± SD of three independent experiments. ^∗^*P* < 0.05, by one-way ANOVA, Dunnett’s test.

With a single exception, no significant differences in colony morphology were observed between KU strains transformed with the empty vector or plasmids expressing each of the AfuCV41362 ORFs (Figure 9). This result was consistent with the lack of difference in colony morphology between native virus-infected and cured strains. The sole exception was that the KU strain with forced expression of ORF4 exhibited a slightly darker colony color than that of other transformants on the PDA plate. This difference presumably reflected the increased conidial number observed in the ORF4-expressing strain (Figure 12B). However, macro- and microscopic examination revealed that radial growth and conidial formation were decreased in the ORF3-expressing strain compared to the empty vector-bearing KU strain (Figures 12A,B, respectively). Microscopic examination also revealed that the numbers of swollen conidia were increased (Figure 12C) and mycelial growth was reduced (Figure 12D) in transformants expressing ORF1.

**FIGURE 13:**
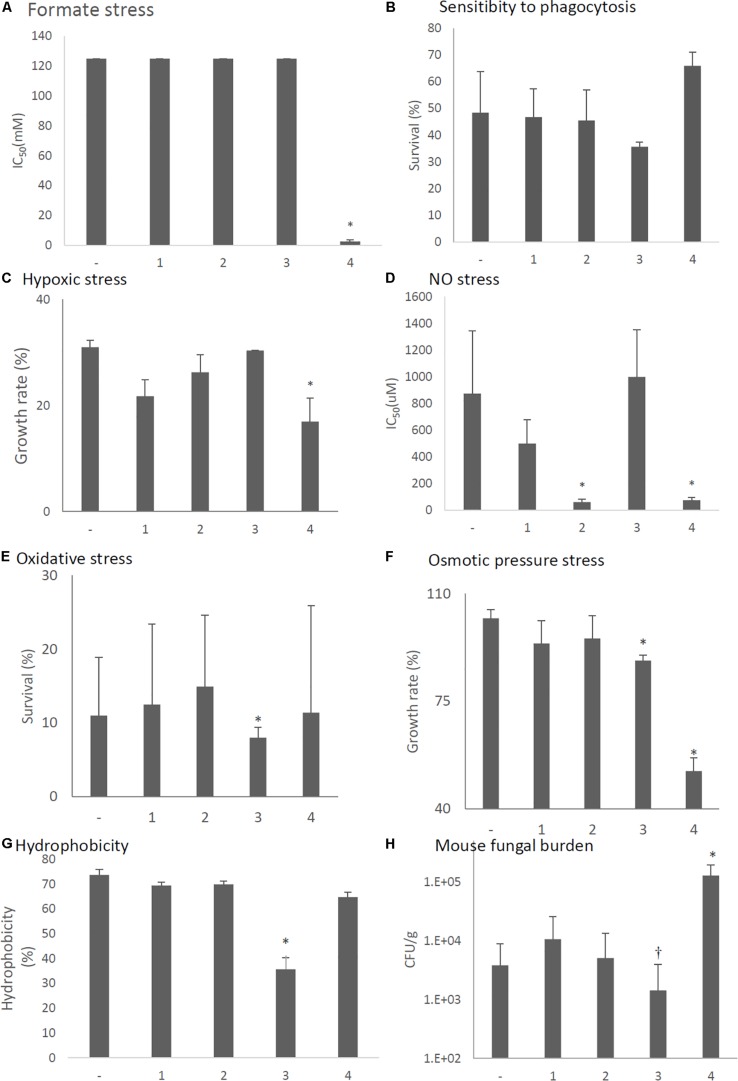
Comparisons of tolerance to stresses, response of mammalian cells, and virulence of ORF-expressing strains. -, KU strain carrying an empty vector. 1–4, KU strains with forced expression of each ORF. The labels are common from graphs **(A)** to **(G)**. **(A)** IC_50_ of the strains under formate stress conditions (RPMI 1640 medium). **(B)** Sensitivity of the *A. fumigatus* strains to phagocytosis by J774A.1, a murine macrophage cell line. Techniques are described in the section “Materials and Methods.” **(C)** Growth of the fungal strains under hypoxic stress conditions. **(D)** IC_50_ of the strains under NO stress conditions (NOC-18 in RPMI 1640 medium). **(E)** Effect of hydrogen peroxide on the growth of the fungal strains. **(F)** Growth of the fungal strains under osmotic stress conditions. **(G)** Hydrophobicity. **(H)** The number of colonies per gram of lung tissue isolated from mice at 72 h after fungal infection. Data are presented as mean ± SD using four mice per group. ^∗^*P* < 0.05, ^†^*P* < 0.15 (nominally not statistically significant). The labels are common from graphs **(A)** to **(G)**. For graphs **(B–G)**, data are presented as mean ± SD of three independent experiments. ^∗^*P* < 0.05, by one-way ANOVA, Dunnett’s test. The data without “^∗^” were NS in comparison to control (KU strain carrying an empty vector).

Stress tolerances were decreased in strains with forced expression of ORF2 (NO stress, Figure 13D), ORF3 (oxidative and osmotic stresses, Figures 13E,F), and ORF4 (formate, hypoxic, NO, and osmotic pressure stresses, Figures 13A,C,D,F). *In vitro* assays revealed that the survival rate of the fungi in co-culture with J774A.1 mouse macrophages slightly decreased in the ORF3-expressing strain and increased in the ORF4-expressing strain (Figure 13B). Hydrophobicity of conidia were reduced in the ORF3-expressing strain (Figure 13G). The effects of ORF-expressing and empty-vector KU strains on virulence were then tested in immunosuppressed ICR mice. As shown in Figure 13G, we observed a nominal (but statistically insignificant; *P* < 0.15) decrease in lung fungal burden (CFU/g) 3 days after infection with the ORF3-expressing strain compared to empty vector control. At the same time point, the lung fungal burden was significantly increased in the ORF4-expressing strain (Figure 13H, *P* < 0.05).

Results for the AfuCV41362-infected (IFM 41362 and KU background) strains and ORF-expressing KU strains are summarized in Supplementary Table S3.

## Discussion

The study of mycoviruses has the potential to yield clinically useful information, as many mycoviruses are known to induce hypovirulence in their fungal hosts. Although several mycoviruses are associated with latent infection of human pathogenic fungi (Jamal et al., 2010; Özkan et al., 2017), to our knowledge there have been no reports of mycovirus-mediated reduction or elimination of fungal pathogenicity in vertebrate infection models. The present study newly identified that AfuCV41362, a dsRNA mycovirus carried by *A. fumigatus* strain IFM 41362, causes hypovirulence of this human pathogenic fungus in a mouse infection model. We believe that this work represents the first report of mycovirus-mediated hypovirulence in a mammalian invasive fungal infection.

### Virus Genome

The genome of AfuCV41362 consists of four dsRNAs, with each dsRNA containing a single ORF. The protein encoded by AfuCV41362 ORF1 showed the highest amino acid sequence similarity (95%) with the RdRp protein encoded by AnV-1816, a virus that infects *A. nidulans* (Hammond et al., 2008a). However, further comparison to AnV-1816 is precluded, as additional AnV-1816 dsRNA sequences have not been published.

Each of the proteins encoded by ORFs 1–4 had high amino acid sequence similarities (47–75%) with the corresponding proteins encoded by the PjCV1 and BdCV-1 mycoviruses. The latter mycovirus was identified from *B. dothidea*, a plant pathogenic fungus (Wang L. et al., 2014). At present, the functions of the proteins encoded by ORF2 and ORF4 are unknown. A homology search suggested that the AfuCV41362 ORF3 protein shared sequence similarity with known capsid proteins. This inferred capsid protein function is consistent with the observation that an immunologically related protein of the same size as the predicted ORF3 protein was detected in VLPs isolated from the virus-infected strain.

The sequences of 5′- and 3′-UTRs were conserved among all four of the AfuCV41362 dsRNAs identified in the present study. This conservation of UTR sequences is a common feature of RNA viruses with multipartite and multi-component genomes (Mertens and Sangar, 1985; Attoui et al., 1997). This result suggests that AfuCV41362 consists of four genomic dsRNA segments that replicate separately as a multipartite dsRNA mycovirus. On the basis of dsRNA number and RdRp sequence similarity, the AfuCV41362 virus can be assigned to the genus Chrysoviridae, cluster II. This is also consistent with the absence of CAA repeats at the 5′-UTRs of the viral genomic dsRNAs, as Chrysoviruses characteristically lack these repeats (Gallie and Walbot, 1992).

### Effects of Virus Infection on Fungal Phenotype

Presence of the AfuCV41362 virus did not alter the colony morphology of the *A. fumigatus* host fungus. On the other hand, conidiophores were smaller in the virus-infected strain than in the virus-free strain, resulting in decreased conidial number. Infecting immunocompromised mice with the virus-infected and the virus-free strains revealed that AfuCV41362 infection reduced *A. fumigatus* virulence in mice. We suggest that AfuCV41362 impairs the survival of conidia inhabiting lung tissue, as virus-infected conidia had reduced tolerance to environmental stresses. Delayed germination and suppression of hyphal elongation by the virus might have caused a reduction in fungal burden in the lung, and further decreased the invasiveness reducing tolerances against various stresses pleiotropically.

AfuCV41362 infection also appears to decrease fungal proliferation, which may also contribute to its hypovirulent effects. Comparison of the lung fungal burden 3 days after infection with the virus-infected or virus-free strains revealed that AfuCV41362 suppressed proliferation of the fungal host in the infected lung. Examination of *in vitro* cultures at 6 h of growth indicated that AfuCV41362 suppressed or delayed swelling of *A. fumigatus* conidia. AfuCV41362 also impaired mycelial growth, as demonstrated by histological observations of infected mouse lungs.

Importantly, the phenotypic effects of AfuCV41362 infection were also observed in the natively virus-free *A. fumigatus* KU strain after infection with AfuCV41362. Specifically, mycovirus-infected KU fungus demonstrated reduced mycelial growth, impaired stress tolerance, and decreased virulence *in vivo* compared with the virus-free KU strain. Taken together, these findings suggest that AfuCV41362 suppresses *A. fumigatus* virulence by reducing stress tolerance and impairing mycelial growth in the mouse lung.

### RNA Sequence Analysis of Fungi

RNA sequencing experiments were performed on infected and cured *A. fumigatus* to identify virus-induced fungal genes potentially related virus-induced hypovirulence. The most potently down-regulated transcripts in virus-infected fungi were *Acia*/*Fdh*. The *fdh* gene transcript is up-regulated in *A. fumigatus* conidia upon exposure to neutrophils under swelling conditions (Sugui et al., 2008). In *Candida albicans*, *Fdh1p* expression is increased after cells have been phagocytosed by macrophages (Prigneau et al., 2003). Fdh catalyzes oxidation of formate to CO_2_. Formate, an indirect product of the glyoxylate cycle, is generated via decarboxylation of oxalate, and is toxic to fungus. Fdh1p regulates formate detoxification in *C. albicans* and *A. fumigatus* cells that have been phagocytized by human immunocytes (Prigneau et al., 2003; Sugui et al., 2008). Thus, we propose that down-regulation of *fdh* in the virus-infected strain may render the pathogen less resistant to the mammalian host defense system. Although tolerance to formate stress was indeed reduced by viral infection, presumably through the function of ORF4, sensitivity to phagocytosis was not affected. Viral infection-mediated reduction of formate stress tolerance may contribute to hypovirulence through mechanisms other than phagocytosis resistance.

During invasive pulmonary aspergillosis, *A. fumigatus* is exposed to a range of *in vivo* microenvironments, including hypoxia (Grahl et al., 2011), and the ability of *A. fumigatus* to adapt to hypoxia is essential for fungal virulence (Grahl et al., 2012). Interestingly, half of the genes down-regulated in AfuCV41362-infected fungus during germination (marked “^∗^” in Supplementary Table S2A) are known to be up-regulated during *A. fumigatus* mycelial growth under hypoxia (Kroll et al., 2014). These loci included genes encoding enzymes involved in NAD^+^ regeneration (FAD-dependent oxidoreductase *frdA*), ethanol fermentation-related genes (pyruvate decarboxylase *pdcA*), alcohol dehydrogenase (*alcC*), and FAD-dependent fatty acid hydratase (myosin-cross-reactive antigen, MCRA, Rosberg-Cody et al., 2011).

Hypoxic response genes are regulated in part by the sterol regulatory element-binding protein (SREBP) SrbA (Willger et al., 2008) and its proteolytic activators RbdA (Vaknin et al., 2016) and DscA-DscD, which are components of the Golgi E3 ligase complex (Willger et al., 2012). However, virus infection did not affect expression of *SrbA or DscA-DscD* during germination. On the other hand, genes regulated by SrbA were down-regulated in the virus-infected strain, including the ergosterol biosynthesis gene *erg11A*/*cyp51A* (Blosser and Cramer, 2012) and the siderophore biosynthesis gene *ftrA*.

Mammalian macrophages and neutrophils generate NO and other reactive nitrogen intermediates that may induce cell death in invading pathogenic fungi. In *Cryptococcus neoformans*, genes encoding *S*-nitrosoglutathione reductase (*gno1*) and flavohemoglobin (*fhb1*) are involved in detoxification of NO radicals, and are essential for fungal virulence (de Jesús-Berríos et al., 2003). In the present study, viral infection reduced tolerance to NO stress, and many NO detoxification genes were down-regulated in the virus-infected strain during germination. These genes included the nitrate transporter *crnA* (2.06-fold reduction), the reactive nitrogen species detoxification flavohemoprotein *fhpA* (153.7-fold reduction), the nitrate reductase *niaD* (36.1-fold reduction), and the nitrogen metabolism nitrite reductase *niiA* (81.7-fold reduction). Contrastingly, transcript levels of the *S*-nitrosoglutathione reductase *gnoA* were not significantly altered by virus infection. However, a previous study suggested that the ability to detoxify host-derived reactive nitrogen intermediates is not essential for the virulence of *A. fumigatus* in the mouse infection model (Lapp et al., 2014). Therefore, the role of AfuCV41362-induced down-regulation of NO detoxification gene transcripts in fungal hypovirulence has not yet been fully elucidated.

Host macrophages and neutrophils also produce high levels of reactive oxygen species (ROS), which is toxic to *A. fumigatus* (Brown et al., 2009). Multiple genes involved in defense against ROS in *A. fumigatus* has been characterized, and some genes were reduced by viral infection during germination. These genes included the sensors of oxidative stress, TcsC (1.89-fold reduction) and PhkB (1.99-fold reduction) (Chapeland-Leclerc et al., 2015), stress response pathway genes YpdA (5.38-fold reduction), PbsB (1.74-fold reduction), and SakA (1.34-fold reduction, Kim et al., 2012), and transcription factor AFUA_4G08120. For comparison, osmotic stress sensor gene sharing the stress response pathway with oxidative stress was also down-regulated (MsbA. 1.87-fold reduction and Sho1, 3.2-fold reduction). In the present study, oxidative stress resistance of *A. fumigatus* was suppressed by infection with the virus, due to the action of ORF3 and 4, but there was no change in the resistance of phagocytosis by macrophages. In the host body, neutrophils are more effective than monocytes and macrophages in their ability to kill the fungi (Chauhan et al., 2006). Defection of oxidative stress resistance by virus infection may help prevent to kill the mycelia by ROS released by neutrophil.

As noted above, many transcripts related to metabolism and ATP generation were down-regulated in the virus-infected strain during germination. Other tolerances to environmental stresses, such as oxidative stress and osmotic stress, were also reduced in the virus-infected strain. Therefore, impaired energy metabolism and stress tolerance may be related to virus-induced hypovirulence of *A. fumigatus* in infected animals. Interestingly, genes encoding proteins implicated in fermentation, DNA processing, DNA recombination, and defense-related functions were commonly down-regulated by virus infection in both the germinating and mycelial growth stages (4 h and 6 days; Supplementary Table S2F). Furthermore, genes related to RNA processing, the cell cycle, and the nutrient starvation response were commonly up-regulated by viral infection in both stages (Supplementary Table S2G). We therefore postulate that the AfuCV41362 mycovirus suppresses host DNA recombination and stress responses while enhancing RNA processing to favor replication of the mycovirus genome. Özkan et al. (2017) reported that introduction of a mycovirus into an *A. fumigatus* host resulted in silencing of host fungal genes that shared sequence homology with the viral genome. The potential mechanisms for mycovirus regulation of host gene transcription remain incompletely understood, and are an important topic for future studies.

Increased viral RNA expression during germination is presumed to adversely affect hyphal growth in *A. fumigatus*. Consistent with this notion, the *A. fumigatus* transcriptome differed dramatically between virus-infected and virus-free strains during germination, while this difference was less pronounced at the hyphal stage of growth.

### Functions of Individual Viral ORFs

To begin to elucidate the role of each viral operation, we induced ectopic expression of individual viral ORFs in the virus-free KU strain. ORF1 has sequence similarity to AsV1816, a partially characterized mycovirus isolated from *Aspergillus niger*. Notably, AsV1816 suppresses RNA silencing through a mechanism mediated by changes in the levels of small interfering RNAs (Hammond et al., 2008a). RNA silencing is an internal defense mechanism that protects the genome from invasion by mobile genetic elements such as viruses (Agrawal et al., 2003). The presence of mycovirus-derived siRNAs impairs the antiviral defense system in fungi (Hammond et al., 2008b; Zhang et al., 2008; Himeno et al., 2010; Özkan et al., 2017). If AfuCV41362 also suppresses host RNA silencing, it is predicted that this function would be relevant to hypovirulence. However, in the present study, viral infection did not affect expression of the RNA silencing-related genes Dicer and Argonaute (Supplementary Table S2H). Our present data demonstrated that ectopic expression of ORF1 facilitated germination, but decreased mycelial growth (dry weight), suggesting that the RdRp enzyme may have affected fungal growth through a mechanism unrelated to RNA silencing.

Ectopic expression of the functionally unknown ORF2 significantly decreased fungal tolerance to NO stress. Notably, forced expression of ORF3 caused reduced radial growth, decreased conidial number, and impaired oxidative and osmotic stress tolerances. Furthermore, ORF3-expressing *A. fumigatus* had modestly decreased virulence in the mouse infection model. Because the ORF3 protein has sequence similarity with the capsid proteins of other mycoviruses, we speculate that the protein encoded by ORF3 localizes to the viral surface, potentially influencing the accumulation of fungal proteins or nucleic acids. These proposed interactions will be addressed in future work.

Ectopic expression of ORF4 had the most significant effect on fungal virulence, but was also controversial to presently available knowledge. Ectopic expression of the ORF significantly reduced conidia stress tolerances *in vitro*, while enhancing fungal burden in mouse lung *in vivo*. The mechanism for this unexpected effect is currently unknown, and will be the topic of exciting future investigations.

Thus, ORF3 had the ability to reduce spores, ORF4 reduced formate and hypoxic stress resistances, and ORF2, 3, and 4 reduced other stress resistance. On the other hand, ectopic expression of individual ORFs did not fully reproduce the hypovirulent phenotype of native AfuCV41362 viral infection (Supplementary Table S3). We suspect that cooperative expression of the other mycoviral ORFs such as ORF 3 and 4 may have synergistic effects on fungal hypovirulence, a hypothesis that will need to be addressed experimentally in future studies.

Further research will be needed to identify the mycovirus genes associated with *A. fumigatus* hypovirulence, along with the underlying mechanisms of these effects. Because the new mycovirus discovered in this study has the highest viral gene expression at the time of germination and has the ability to suppress various stress tolerances in the host *A. fumigatus*, such work is expected to be of great value for both basic research and the development of novel therapeutic agents targeting fungal infection.

## Materials and Methods

### Fungal Strains and Culture Conditions

*Aspergillus fumigatus* strain IFM 41362 was isolated from a patient with neonatal systemic aspergillosis in Japan and stored in the Medical Mycology Research Center of Chiba University, Japan. *A. fumigatus* strain KU (Afs35, aku70, Krappmann et al., 2006) was obtained from the Fungal Genetics Stock Center (Kansas State University, KS, United States). Strains were cultured at 37°C in potato dextrose broth (PDB; BD Biosciences) medium. Conidia were harvested from strains cultured at 37°C on potato dextrose agar (PDA) plates (BD Biosciences).

### Mammalian Cell Lines

The mouse macrophage-like cell line J774A.1 was obtained from Riken BRC Cell Bank and maintained in RPMI 1640 medium supplemented with 10% fetal bovine serum (Gibco), 100 mg/L streptomycin (Sigma-Aldrich, St. Louis, MO, United States), and 16 mg/L penicillin (Sigma). Cells were cultured at 37°C in a humidified 5% CO_2_ incubator.

### Extraction, Identification, and Sequencing of dsRNA

IFM 41362 mycelia were collected from 14-day-old cultures (grown at 37°C in PDB) and stored at −80°C until use. dsRNA was extracted and purified from the mycelia using a cellulose chromatography method followed by digestion with DNase 1 (TaKaRa Bio) and S1 nuclease (TaKaRa Bio), as described previously (Okada et al., 2015). dsRNA was electrophoresed in 5% (wt/vol) polyacrylamide gels and stained with ethidium bromide for visualization. To identify dsRNA sequences, dsRNAs were electrophoresed on a 1% agarose gel for 16 h. Separated bands were individually excised and subsequently purified with a Zymoclean Gel RNA Recovery Kit (ZymoResearch). Purified dsRNAs were subjected to double-stranded cDNA synthesis using a cDNA Synthesis kit (Roche). Adaptors, obtained as Set A from the TruSeq RNA Sample Preparation Kit, v. 2 (Illumina), were added to the resulting double-stranded cDNAs, and the sequences of these cDNAs were determined using a MiSeq genome sequencer (Illumina). The resulting paired-end reads were assembled using the CLC genomics workbench, ver. 6.5.1 (Filgen). The assembled contigs (>1,000 nt long) were analyzed by a BLASTN (Ver. 2.2.28+) search of the DDBJ/GenBank/EMBL databases, and sequences with similarity to those of previously reported mycoviruses were considered candidate components of the novel *A. fumigatus* mycovirus. Based on the sequences of the contigs, we designed primers (Supplementary Table S4) and confirmed the viral genome sequences using a primer-walking strategy. The 5′- and 3′-terminal sequences of the dsRNAs were determined using a 5′-RACE kit (5′-Full RACE Core set; TaKaRa Bio). Genome sequences for AfuCV41362 were deposited in DDBJ/GenBank/EMBL as accession numbers LC350094-LC350097.

The sequences of previously reported mycoviruses and related genes were retrieved from the NCBI^1^ databases and used for comparative analysis. Multiple sequence alignments were conducted using the Clustal X program (Ver. 2.0; Larkin et al., 2007) to identify conserved motifs. A phylogenetic tree was constructed based on the amino acid sequence of the putative and known RdRps and capsid proteins using the MEGA 7 program Ver. 7.0 (Kumar et al., 2016). Bootstrapping tests were conducted on 1000 re-samplings for the NJ tree.

### Northern Blot

Northern hybridization was performed to verify the authenticity of the cDNA sequence generated from the dsRNAs. dsRNA extracted from strain IFM 41362 was separated by agarose (1%) – formaldehyde (2.2 M) gel electrophoresis and transferred to an Immobilon-N membrane (Millipore, United States). Four DNA probes were designed on the basis of the AfuCV41362 genome sequence, and generated by RT-PCR using the primer sets in Supplementary Table S4. The probes were labeled with digoxigenin by AlkPhos Direct (GE Healthcare) and together hybridized to the dsRNA blotted on the nylon membrane. The hybridization signals were detected by enzymatic immunoassay using the reagents in the AlkPhos Direct kit according to the manufacturer’s procedures. Signals were visualized using a luminescent image analyzer (LAS-1000 mini; FUJIFILM).

### Purification of Virus Particles

Strains were cultured at 37°C in PDB medium for 7 days, and frozen mycelia (10 g) were pulverized using a mortar and pestle and suspended in 100 mL 0.1 M sodium phosphate buffer (pH 7.4) containing 0.2 M KCl. This suspension was shaken at 3,000 rpm for 30 min and subsequently centrifuged at 10,000 × *g* for 30 min. The resulting supernatant was successively filtered through 0.45 μm and then 0.2 μm bottle-top filters (IWAKI, Japan). Polyethylene glycol (PEG6000, Merck, Germany) and NaCl were added to the supernatant to final concentrations of 8 (w/v) and 1% (w/v), respectively. After overnight incubation at 4°C, the precipitate was collected by centrifugation (10,000 × *g*) at 4°C for 20 min, and the pellet was re-suspended overnight in 1 mL 0.05 M sodium phosphate buffer (pH 7.0) at 4°C. The suspension was then applied to a column of HiTrap NHS-activated HP (GE Healthcare) and conjugated with the anti-ORF3 protein antibody described below. VLPs were eluted with elution buffer, stained with 2% uranyl acetate, and examined by TEM (JEM-1400, JEOL, Tokyo, Japan).

### SDS-PAGE and Western Blotting Analysis of Viral Proteins

Proteins extracted from the VLPs were analyzed by 10–14% SDS-PAGE. After electrophoresis, the gels were stained with Coomassie Brilliant Blue R-250 (Bio-safe CBB, Bio-Rad).

Rabbit polyclonal antibody was raised against a synthesized peptide with a partial amino acid sequence of ORF3, RIADPDEQLDEAC (Eurofins Co., Brussels, Belgium), selected on the basis of a high antigenicity score. An additional portion of the VLP extract also was resolved by 10–14% SDS-PAGE and transferred to a nitrocellulose membrane (Immobilon-P membrane, Millipore, United States). The membrane was blocked using 5% bovine serum albumin in TBS-Tween 20 (TBS-T) for 1 h, washed with TBS-T, incubated with the anti-ORF3 protein antibody overnight at 4°C, washed again with TBS-T, and subsequently incubated with horseradish peroxidase-conjugated secondary antibody (Jackson ImmunoResearch Lab, Inc.) for 1 h at room temperature. The viral ORF3 protein was detected using an enhanced chemiluminescent reagent system (Thermo Scientific) on an LAS-1000 mini.

### Relative Quantification of Viral RNA Expression Levels by Real-Time RT-PCR

For the synchronized induction of asexual development, conidia (10^5^ conidia/mL) were cultivated at 37°C in 20 mL PDB medium for 7 days, and conidia-free mycelia were harvested using Miracloth (Merck, Germany), washed with distilled water, and transferred onto PDA plates. The plates were then incubated at 37°C for specified times. The start of this plate incubation was referred to as 0 h, and mycelia were harvested at 0, 6, 12, 24, and 48 h time points.

Total RNA was isolated from *A. fumigatus* mycelium or conidia using an RNeasy Mini kit (Qiagen). Synthesis of cDNA from the total RNA was conducted with ReverTraAce using random primers (Toyobo, Japan). Subsequently, real-time PCR was conducted in 96-well plates with 20 μL reaction volumes containing THUNDERBIRD SYBR qPCR Mix (Toyobo, Japan). The samples were subjected to denaturation at 95°C for 30 s, followed by 40 cycles of amplification (95°C for 15 s, 60°C for 30 s) using a LightCycler 96 (Roche). Expression levels of viral RNA were normalized to the level of the constitutively expressed *A. fumigatus tef-1* gene, which served as an internal control (Gravelat et al., 2008). Primer sets are shown in Supplementary Table S4.

### Curing AfuCV41362 Virus From *A. fumigatus* IFM 41362

Single spore isolation was performed using micromanipulation. Conidia were collected from IFM 41362 grown on PDA. Single spores were isolated and seeded on new individual PDA plates and cultured for 14 days. Newly obtained spores were grown in PDB for further experiments. The presence or absence of AfuCV41362 was confirmed by agarose gel electrophoresis of purified dsRNA, and by staining fixed fungal cultures with an anti-dsRNA mouse monoclonal antibody followed by detection using an Alexa488-labeled goat anti-mouse IgG secondary antibody (Thermo Fisher Scientific Co.).

### Morphological Studies

Fungal growth was evaluated by quantifying colony diameter. About 200 conidia of *A. fumigatus* strains were point inoculated on PDA medium and incubated in the dark at 37°C for 2 days before colony diameter was measured. Experiments were conducted with three independent replicates. For observation of colony morphology, fungal strains were point inoculated on yeast-glucose minimal medium (0.1% yeast extract – 1% glucose, YGM).

Conidia were harvested in phosphate-buffered saline (PBS, pH 7.4) containing 0.1% Tween 20. The numbers of conidia were determined using a hemocytometer (Watson Biolab, Japan).

To observe the conidiophores of the virus-infected and virus-free strains, fungi were grown on PDA for 3 days. The resulting agar blocks were fixed using the osmium vapor technique at 4°C for 2 days, dried in a desiccator, metalized with platinum–palladium, and observed by scanning electron microscopy (SEM, S-3400N, HITACHI, Tokyo, Japan).

For measuring the percentage of swelling conidia, conidia (10^6^ conidia/mL) were inoculated in 200 μL YGM on chamber slides (Lab-Tec, Nunc) and incubated at 37°C. Ratios of swelling conidia number to the total number of conidia were determined by microscope observation at 6 h after the start of incubation.

To compare the mycelial growth, dry weights of mycelia at 24 h after starting incubation of conidia in PDB medium.

### Measurement of Stress Responses

#### Nitric and Formate Stresses

Conidia were diluted in RPMI 1640 medium (SIGMA) to 5 × 10^3^ cells/mL, and subsequently treated with different concentrations of NOC-18 (NON-Oate:1-substituted diazen-1-ium-1,2-diolates, Dojindo, Japan) or formic acid (Wako, Japan) at 37°C for 4 days on 96-well plates in the dark. Optical density was measured at 600 nm (OD_600_, iMark Microplate Reader, BIO RAD), and the 50% inhibitory concentration (IC_50_) of each stressor was calculated. The procedure for NO stresses was based on McElhaney-Feser et al. (1998).

#### Hypoxic Stress

To measure growth under hypoxic stress conditions, strains were cultured in an anaerobic culture system using the Anaeropack Kenki (Mitsubishi Gas Chemicals, Tokyo, Japan). A total of 10^6^ conidia of each strain were point-inoculated on YGM in a dish, and the culture dish was placed in an airtight jar with an Anaeropack and incubated at 37°C for 7 days. In the jar, the oxygen concentration was maintained at <1%, and carbon dioxide concentration was maintained at approximately 5%. The growth ratio under each of these stress conditions was calculated by comparing the radial colony diameters on YGM plates with and without hypoxic stress.

#### Oxidative Stress

To measure growth under oxidative stress conditions, 1 × 10^2^ conidia/mL were incubated with 32.6 mM H_2_O_2_ in PDB medium. After incubating for 1 h at 37°C, the 100 μl suspensions were immediately plated onto Sabouraud dextrose agar (SDA) on 9 cm Petri dishes. After incubation for 24 h at 37°C, the numbers of visible colonies were counted. The growth ratio under oxidative stress was calculated by comparing the colony number on SDA for cells plated with and without H_2_O_2_. This procedure was based on Paris et al. (2003).

#### Osmotic Stress

To measure fungal growth under osmotic stress conditions, 10^6^ conidia of each strain were point-inoculated on YGM containing 0.8 M NaCl. After incubation for 72 h at 37°C, colony diameter was measured.

#### Hydrophobicity Measurement

Conidia were harvested, washed twice, and suspended in PBS at OD_600_ = 0.4. The conidial suspension was treated with excess xylene (2.5:1, v/v), mixed vigorously for 2 min, and allowed to settle for 20 min. OD_600_ of the aqueous phase was determined and the relative hydrophobicity was calculated as described previously (Reeves et al., 2006).

### *In vivo* Virulence Assay

The mouse model of pulmonary aspergillosis was performed according to Wang D.-N. et al. (2014) with slight modifications. Male ICR mice were obtained from the Takasugi Experimental Animals Supply Co., Ltd. (Japan), and were housed under sterile conditions for acclimation. Six-week-old mice were immunosuppressed by subcutaneous injection of 200 mg/kg cyclophosphamide (Shionogi Pharmaceutics Co., Ltd., Osaka, Japan) on days −2, 0, and 2. Sterile drinking water containing 300 mg/L tetracycline hydrochloride was provided beginning on day −7. On days 0 and 1, each mouse was infected intratracheally with 5 × 10^7^ conidia suspended in 20 μL PBS – 0.01% Tween 20. Twenty mice per group were used. Morbidity and mortality were monitored for up to 14 days, and a Kaplan–Meier survival analysis with log rank test was used for comparison among the groups. To determine lung fungal burden, immunosuppressed mice were infected by the same method. Five mice from each group were sacrificed 72 h post-infection. The lung tissues were weighed, homogenized, and serially diluted onto PDA, and the resulting plates were incubated at 37°C. After 24 h, the number of colonies per gram of lung tissue (CFU/g) was determined. For histopathological examination, lung tissue was dissected from animals of each group at 72 h post-infection, fixed in 10% (vol/vol) formaldehyde, and stained with hematoxylin and eosin (H&E) and Grocott’s methenamine silver (GMS). Mycelial length was measured using SMileView software (JEOL).

### Ethics Statement

All animal studies were conducted in accordance with the “Guidelines for Proper Conduct of Animal Experiments” formulated by the Science Council of Japan on 1 June 2006^2^. All animal protocols used in this study were approved by the Institutional Animal Care and Use Committee of Chiba University (Permit Numbers DOU25-206, DOU26-227, DOU 27-35, and DOU28-46). Every effort was made to minimize pain and discomfort in strict accordance with the principles outlined by the “Guidelines.” Animals were clinically monitored at least daily, and humanely euthanized if moribund (defined by lethargy, dyspnea, and weight loss). All sacrifices (both of moribund animals and of surviving animals at the end point) were performed by cervical dislocation.

### *In vitro* Killing Assay by Macrophages

For the *killing assay by macrophages*, J774A.1 murine macrophages were grown to confluence in 6-well plates. Subsequently, 1 × 10^2^ conidia were added to each well, and the plates were incubated at 37°C under 5% CO_2_ to induce phagocytosis. After 2 h of co-culture to allow conidial adherence, cell monolayers were rinsed with PBS to eliminate conidia not adhered to the cells, 2 mL of culture medium was added to each well and the plates were incubated for another 5 h to permit phagocytosis. The samples with 0 h conidia attached to the cells were used as a control. Subsequently, cell monolayers were rinsed with PBS, overlaid with SDA, and incubated at 37°C for 20 h. The phagocytosis rate was determined as the number of monocolonies expressed as the percentage of the control (5 h CFU/0 h CFU).

### RNA-Seq

Total RNA was extracted from fungal cells using an RNeasy Mini Kit (Qiagen) and treated with DNase I (TaKaRa, Japan). Polyadenylated mRNA was then extracted from total RNA and fragmented using a TruSeq RNA Sample Preparation Kit v2-Set A (Illumina). A 200- to 300-nt size selection was performed, and the RNA was then converted into an Illumina sequencing library according to the manufacturer’s protocol. Libraries were sequenced on a MiSeq sequencer (Illumina) as 50-bp single-end reads. The CLC genomics workbench, ver. 6.5.1 (Filgen), was used to analyze the sequence results. Transcripts were categorized using the MIPS Functional Catalog^3^. The sequence data have been deposited in the DDBJ/EMBL/GenBank database under the GEO accession number PRJDB9005.

### Transmission of Mycovirus to Virus-Free KU Strain via Protoplast Fusion

The protoplast fusion method was used to infect virus-free KU strains with the mycovirus as described previously (Kanematsu et al., 2004; Lee et al., 2011). Briefly, KU strains transformed with the empty vector pCB1004, which carries the hygromycin B resistant marker (*hph*), and *A. fumigatus* strains infected with AfuCV41362 (IFM 41362) were individually grown in PDB medium at 37°C for 1 day. Protoplasts were prepared separately from the two specimens by treating with TF solution 1 [50 mM maleic acid (pH 5.5 with NaOH), 0.6 M (NH_4_)_2_SO_4_], 5 mg/ml Yatalase (TaKaRa Bio, Shiga, Japan), and 5 mg/ml lysing enzyme from *Trichoderma harzianum* (Sigma-Aldrich, St. Louis, MO, United States) at 30°C for 3 h. Equal volumes of the two protoplast suspensions were mixed and placed on ice for 30 min. PEG solution (60% PEG 3350, 10 mM MOPS pH 7.0, and 10 mM CaCl_2_, final concentrations) was then added to the protoplast suspension, and the mixture was incubated at 30°C for 30 min. Protoplast fusants were selected on PDA plates containing 50 mg/ml hygromycin B.

### Generation of Viral ORF-Expressing Strains

Fungal strains expressing the individual viral ORFs were generated by transforming the KU *A. fumigatus* strain with recombinant derivatives of plasmid pCB1004. First, the coding regions of viral ORF1 to 4 were individually PCR-amplified using mycovirus AfuCV41362 dsRNAs as templates, and corresponding primer sets containing appropriate restriction sites (Supplementary Table S4). Each fragment was then digested with the corresponding restriction enzymes and ligated into the appropriate cloning sites of pCB1004 (Carroll et al., 1994). pCB1004 contains the *A. fumigatus gpdA* promoter [gpdA(p)], the *A. fumigatus gpdA* terminator [gpdA (t)], and the *A. fumigatus hph* selection marker. Integration of the expression vectors into the genome was confirmed by PCR using the primer sets listed in Supplementary Table S4. Expression of viral ORFs was confirmed by quantitative RT-PCR. The empty vector was used as a negative control.

## Data Availability Statement

Publicly available datasets were analyzed in this study. This data can be found here: Sequence files of AfuCV41362 segments 1–4 are available from the DDBJ database (accession numbers: LC350094–LC350097; https://www.ddbj.nig.ac.jp/index-e.html). The RNA-seq data have been deposited in the DDBJ/EMBL/GenBank database under the GEO accession number PRJDB9005.

## Ethics Statement

The animal study was reviewed and approved by the Institutional Animal Care and Use Committee of Chiba University (Permit Numbers DOU25-206, DOU26-227, DOU 27-35, and DOU28-46).

## Author Contributions

AT-N and HM designed the experiments. AT-N, ES, MY, SU, KS, HC, and KK performed the experiments. AT-N, ES, and TG analyzed the data. AT-N and TG wrote the manuscript.

## Conflict of Interest

The authors declare that the research was conducted in the absence of any commercial or financial relationships that could be construed as a potential conflict of interest.

## Footnotes

1 https://www.ncbi.nlm.nih.gov/

2 http://www.scj.go.jp/en/animal/

3 https://elbe.hki-jena.de/fungifun/

## Supplementary Material

FIGURE S1 Multiple alignments of 5′- and 3′-untranslated regions (UTRs) of the four identified dsRNA segments with respect to the coding strand (ORF sense strand). Asterisks indicate identical nucleic acids, and periods indicate conserved nucleic acids.

FIGURE S2 Comparison of dsRNA bands between the AfuCV41362-infected strain (lane V) and the virus-free strain (lane F) by agarose gel-electrophoresis. Lane M, molecular weight marker.

FIGURE S3 Functional catalog analysis of down-regulated **(A)** and up-regulated **(B)** genes at 4 h in swelling stage, and down-regulated **(C)** and up-regulated **(D)** genes on 6-day in conidia-forming stage. The analysis was performed using FungiFun web application (Priebe et al., 2011). Genes that have more than two-fold reductions in between the virus-infected and virus-free strains are analyzed. Detailed data underlying Supplementary Table S2 were attached as MS Excel file of Supplementary Table S2.

FIGURE S4 Agarose-gel electrophoresis of RNA fractions isolated from virus-free KU strain (−) and virus-infected strains (w). M, molecular weight marker.

TABLE S1 **(A,B)** Information for the virus isolates used for sequence alignment and phylogenetic analysis of RdRps and CPs in Figure 2, respectively.

TABLE S2 Total and integrated information of up- or down-regulated genes by virus infection. **(A,B)** Lists of the **(A)** down-regulated and **(B)** up-regulated *A. fumigatus* genes in the AfuCV41362 virus-infected strain in comparison to the virus-free strain at 4 h (swelling stage). ^∗^ indicates hypoxia-induced genes reported in previous work (Kroll et al., 2014). **(C,D)** Lists of the **(C)** down-regulated and **(D)** up-regulated *A. fumigatus* genes in the AfuCV41362 virus-infected strain in comparison to the virus-free strain on day 6 (conidia-forming stage). **(E)** Estimated numbers of genes regulated by mycovirus infection. **(F,G)** Genes commonly down-regulated and up-regulated, respectively, at 4 h (swelling stage; **F**) and day 6 (conidia-forming stage; **G**). The functional catalog data are also provided in **H**. Changes of expression levels in RNA-silencing related, dicer- and argonaute-like genes. Fold reductions are free/virus-infected.

TABLE S3 Summary of results comparing *A. fumigatus* strain infected with the AfuCV41362 virus (native virus) or transformed with plasmid for ectopic expression of each AfuCV41362 ORF (ORF1-4). Effects were assessed during sporulation (upper), during mycelial growth (middle), and as virulence in mouse (bottom). Phenotypic changes at each developmental stage are shown. Red downward arrows are indicative of decrease and black upward are indicative of increase, respectively, in comparison to the respective control (virus-free strain or strain transformed with an empty vector). Effects are indicated only when statistical significance (*P* < 0.05) was achieved (except black downward arrow, where *P* < 0.2). CFU, colony forming unit.

TABLE S4 Primers used in the present study.
