# Supplementary material for: Analysis of an Intrinsic Mycovirus Associated With Reduced Virulence of the Human Pathogenic Fungus Aspergillus fumigatus
Source: Front Microbiol. 2020 Jan 17;10:3045. doi: 10.3389/fmicb.2019.03045 (PMC6978690; doi:10.3389/fmicb.2019.03045)
Supplement: FIGURE S1 — Multiple alignments of 5′- and 3′-untranslated regions (UTRs) of the four identified dsRNA segments with respect to the coding strand (ORF sense strand). Asterisks indicate identical nucleic acids, and periods indicate conserved nucleic acids. [file Data_Sheet_1.PDF]

5’-UTR region

```
Segment 1    1:ACGCAAAAAATAGTAAAAGTGTTTTTGTGAACATCTTTTACTGGGAGGTTTTTTTTTTCT 60
        2    1:GTGCAAAAAAGAGAAAAAGGCGTTTCTC--CTTTTGTAGATGTGACGCGCCACTTTAGAT 58
        3    1:--GCAAAAAAGAGAAAAAAGGAAAA-----AGG-CTTTGGGTCGGCAGGCCAATTGAACA 52
        4    1:--GCAAAAAAGAGAAAAAGGCAAAACCTTTGTA-GTCAAGCTTAGTGCGCCAATTGAACG 57
                 ********.**.****..               ..    . .. . .... ** . .

Segment 1   61:TTCATTTTTGCACACAAGCGTAGAT--CAGCTCGCCCCTGTTAGTTTAGCTCG------- 111
        2   59:TGTACTCTTAGTC-CGATCGAAAGT----GCCGCGTGGCATAGGTGCAGATAAGCAATAC 113
        3   53:GGCGCCCGCGGCGTTTG-CGCAATTGCCCGAAGGGTATTGGAAATTTGTCTA---ACTCA 108
        4   58:CGCTTTCGCGAAAACGGGTTCAATTGCC-GTCAGGCCTAAGTGATATACCAATAGAACAA 116
                ..  ..  .    .   .. *. *  . *   ..         * .. ...   .

Segment 1  111:------------------------------------------------------------ 111
        2  114:ATACCACAGCGGTGACTCTATCTACGCCCGTTCCAGTTGCAGGTCAAGCCGTGACTGGTC 173
        3  109:CAGAACGTTCTG-ACTACACGCCGATCCAACGTGTCTCG-ATCCCTCTCTATTGCAAGC- 165
        4  117:CTGGCTGACGGGTACTTCAGTACGCATCACTGCTTCCGGTAGATCCTATCACCGTAAGCA 176
                          .     .         .          . .   .            .

Segment 1  111:------------------------------------------------------------ 111
        2  174:CAGTGAGCGTCAATTTGACTTCGTTAAGTTACATTGAATAAGGTGAGTTGCTTGCCCTTC 233
        3  166:--TAAGCTGTGATAGAGACATCTTGCCCCGTCAGT------------------------- 198
        4  177:CATAACGACTGAACGATTACGCTTGCATTATCCACCAATCTCAAGACC------------ 224
                        . .         . .       .

Segment 1  111:------------------                                           111
        2  234:GAAGAGGTTGAGTCGAAG                                           251
        3  198:------------------                                           198
        4  224:------------------                                           224
```

3’ UTR region

```
Segment 1 3517:--------------------------------------------------------AAGA 3520
        2 2604:---------TGTGCGCTGGCAGTTTATGGTTGGATCTCCGCTTTACGGTCATGACTAATG 2654
        3 2488:CACCCGCCCTTCACGATGTGGAAGCGACGCTGACCGAGACGCAGAAAAAGGTTTTGACTA 2547
        4 2340:AGCTAGGTTTGCCCGAGGCTGGCTCAAACCAACATCATGCATCGAATAGGACACTGGCAA 2399
                        .   ..  .                          .           .  .

Segment 1 3521:TTGTCGCTCGCGGTTGTAAGGCACGGATCTTA---CGCGCATTTCCA------------- 3564
        2 2655:GTGTAATT-GCAACCCGGCCGCACGGACCTTAG-CCACACGTGGCTAAGGTCGTGTA-AA 2711
        3 2548:CTGGTAAGCTCGACACGGTCGTACGGATCGCAT-TGACGTTTCAGTGCGGTCGTGCA-AA 2605
        4 2400:TAGGTTTGATGAGATCGCAGTCGCGGTTGTGGGGCGGCGCACAATCAGTTTGACGTTCAG 2459
                 .*       .    ..   ...***.... .     *.. .    .   .   .   .

Segment 1 3564:------------------------------------------------------------ 3564
        2 2712:GTAGCACATACGCTGCGGGACGTTTCCCCCAGTTTATGTGTATCCGTCTACTCCAGGCCA 2771
        3 2606:GTTAACAGGTTGCAGCTGTTAGGGTGCAGCACGCTAACGTAATCTAGTCGGCGTCCGGCT 2665
        4 2460:GCTGGTTCTGTGTAGCCCCATCGGCGCGCAGACGCATTGCGACTTCGACGTTTCGGGGTC 2519
               .          .  ..          .        .     .              .

Segment 1 3564:------------------------------------------------------------ 3564
        2 2772:TGTCTCTCCTCAAAGTGTACTCGATGCGGTGCTACTGCTTTTGAGCAGTTGCCGGTCGGT 2831
        3 2666:AGGTTAGGTTAACGGCGTGCTAACTAAGCTTTGGCGCGGCGTGAGCCCGTCAATCCCTCT 2725
        4 2520:GTCTTGCTCTTAGCGCCAAGCTAACGTGACGCCATTGACGTTCAGTGGCGGCAGGTTAGC 2579
                   .    . .  .            .             . ..

Segment 1 3564:--------------------------------                             3564
        2 2832:CCTGCTTTGGCGCATTCCGGCGCGTCAATCCC                             2863
        3 2726:CCG-----------------------------                             2728
        4 2580:CTCGCTTTGGCGCGGCTCGCCGCGTCAATCCC                             2611
               .
```

Figure S1.

Table S1

A. RdRps

Virus name	Abbreviation	GenBank accession no.	Family	Genus
Aspergillus mycovirus 1816	AsV1816	ABX79996	Chrysoviridae	Tentative Chrysovirus
Penicillium janczewski chrysovirus 1	PjCV1	YP_009182332	Chrysoviridae	Tentative Chrysovirus
Botryosphaeria dothidea chrysovirus 1	BdCV1	AGZ84312.1	Chrysoviridae	Tentative Chrysovirus
Aspergillus thermomutatus chrysovirus 1	AthCV1	AWC67507.1	Chrysoviridae	Tentative Chrysovirus
Magnaporthe oryzae chrysovirus 1	MoCV1	AB560761	Chrysoviridae	Tentative Chrysovirus
Tolypocladium cylindrosporum virus 2	TcV2	CBY84993	Chrysoviridae	Tentative Chrysovirus
Fusarium graminearum dsRNA mycovirus 2	FgV2	ADW08802	Chrysoviridae	Tentative Chrysovirus
Fusarium graminearum dsRNA mycovirus China 9	FgVch9	ADU54123	Chrysoviridae	Tentative Chrysovirus
Helminthosporium victoriae 145S virus	HvV145S	YP_052858	Chrysoviridae	Chrysovirus
Amasya cherry disease-associated chrysovirus	ACDCV	YP_001531163	Chrysoviridae	Chrysovirus
Penicillium chrysogenum virus	PcV	YP_392482	Chrysoviridae	Chrysovirus
Aspergillus fumigatus chrysovirus	AfuCV	CAX48749	Chrysoviridae	Chrysovirus
Verticillium dahliae chrysovirus 1	VdCV1	ADG21213.1	Chrysoviridae	Chrysovirus
Cryphonectria nitschkei chrysovirus 1	CnCV1	ACT79258	Chrysoviridae	Chrysovirus
Saccharomyces cerevisiae virus L-BC	ScVLBC	NP_042581	Totiviridae	Totivirus
Saccharomyces cerevisiae virus L-A	ScVLA	NP_620495	Totiviridae	Totivirus
Helminthosporium victoriae virus 190S	Hv190SV	NP_619670	Totiviridae	Victorivirus
Sphaeropsis sapinea RNA virus 1	SsRV1	NP_047558	Totiviridae	Victorivirus

B. CPs

Virus name	Abbreviation	GenBank accession no.	Family	Genus
Penicillium janczewski chrysovirus 1	PjCV1	YP_009182337	Chrysoviridae	Tentative Chrysovirus
Botryosphaeria dothidea chrysovirus 1	BdCV1	YP_009353029.1	Chrysoviridae	Tentative Chrysovirus
Aspergillus thermomutatus chrysovirus 1	AthCV1	AWC67508.1	Chrysoviridae	Tentative Chrysovirus
Magnaporthe oryzae chrysovirus 1	MoCV1	BBG92295.1	Chrysoviridae	Tentative Chrysovirus
Fusarium graminearum mycovirus-China 9	FgVch9	ADU54125.1	Chrysoviridae	Tentative Chrysovirus
Fusarium graminearum dsRNA mycovirus 2	FgV2	ADW08804.1	Chrysoviridae	Tentative Chrysovirus
Helminothosporium victoriae 145S virus	HvV145S	NC005979	Chrysoviridae	Chrysovirus
Amasya cherry disease associated chrysovirus	ACDCV	AJ781165	Chrysoviridae	Chrysovirus
Penicillium chrysogenum virus	PcV	NC007540	Chrysoviridae	Chrysovirus
Aspergillus fumigatus chrysovirus	AfuCV	FN178513	Chrysoviridae	Chrysovirus
Verticillium dahliae chrysovirus 1	VdCV1	YP_009507946	Chrysoviridae	Chrysovirus
Cryphonectria nitschkei virus	CnCV1	GQ290648	Chrysoviridae	Chrysovirus

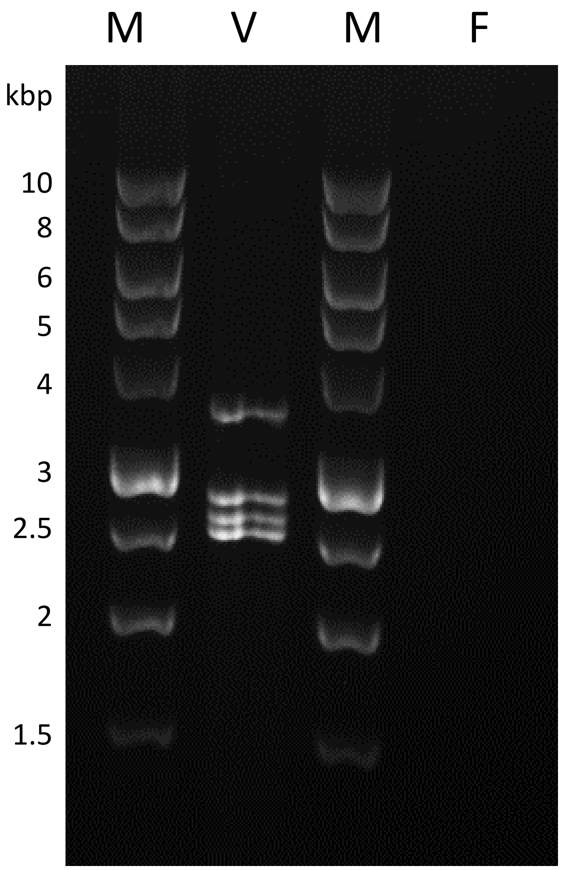

Figure S2

Figure S3.

A. Down-regulated at swelling stage (4 h)

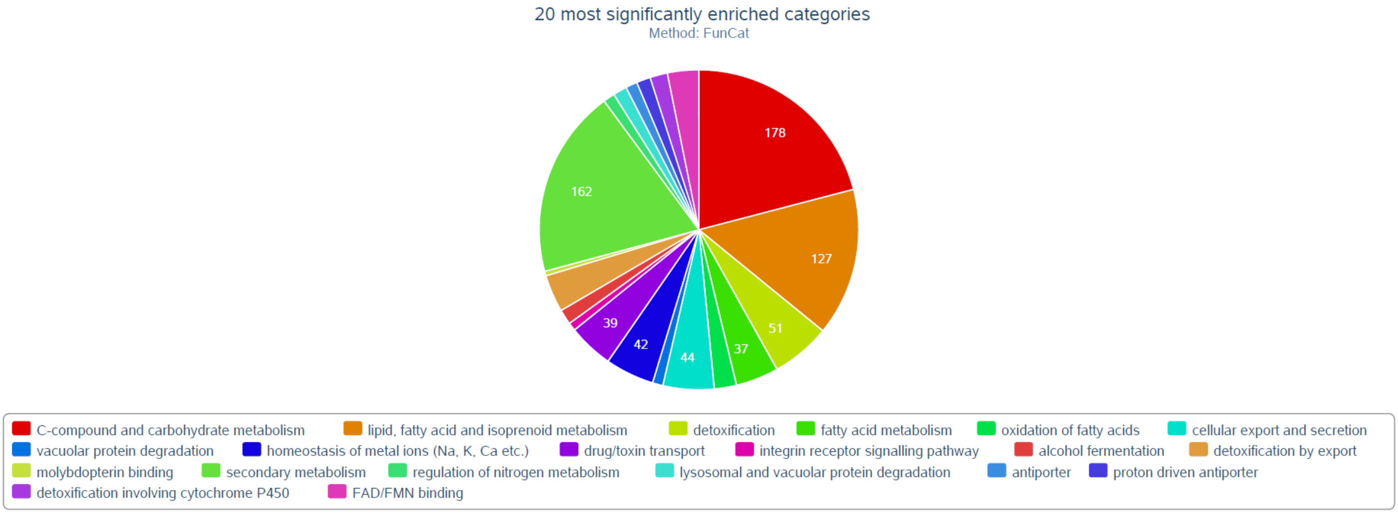

B. Up-regulated at swelling stage (4 h)

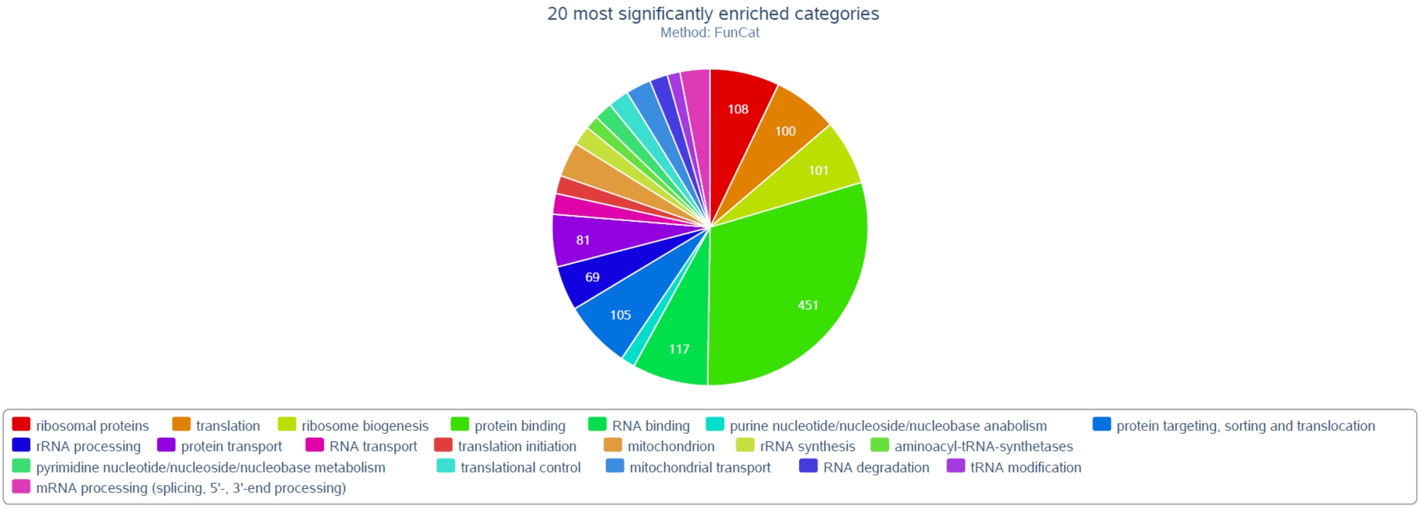

Figure S3.

C. Down-regulated at conidia forming stage (on day 6)

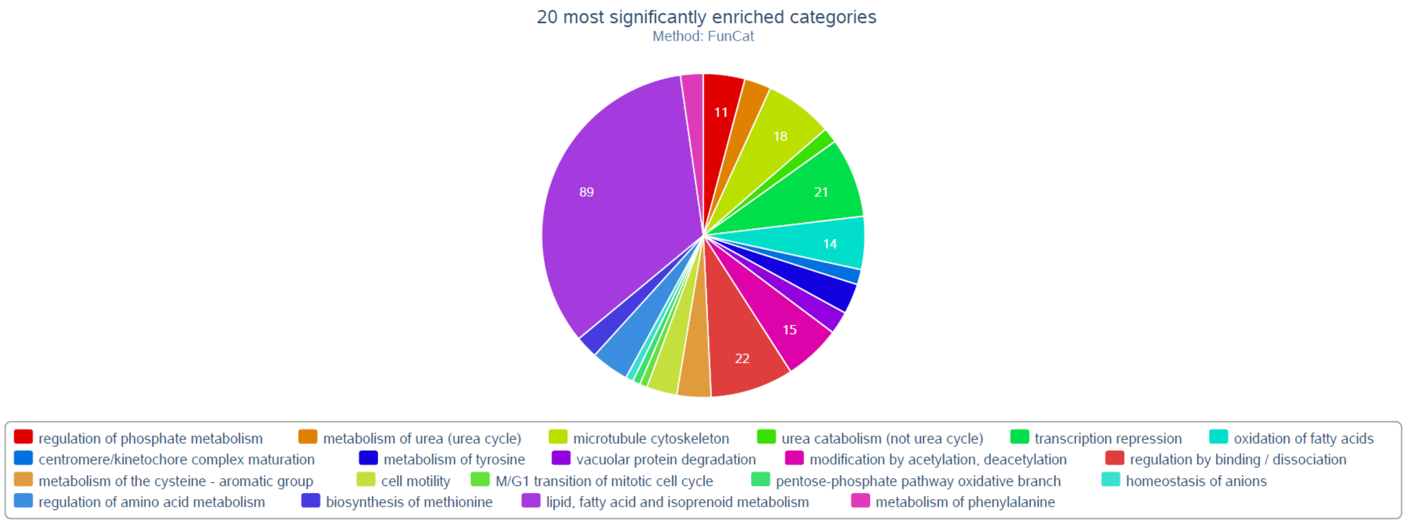

D. Up-regulated at conidia forming stage (on day 6)

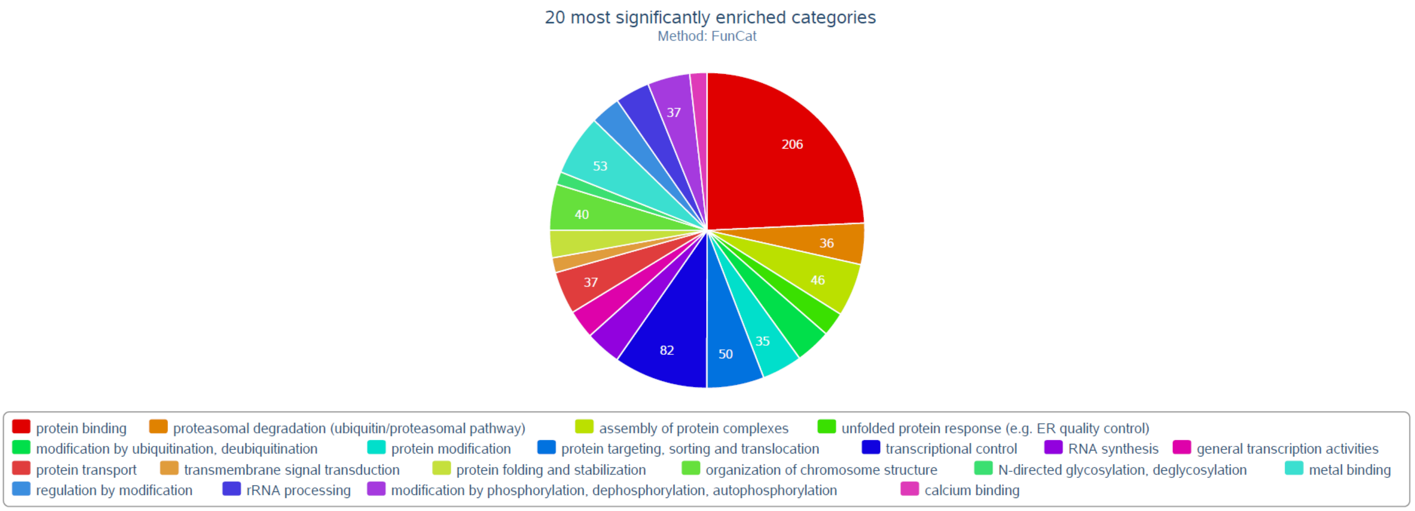

Figure S4.

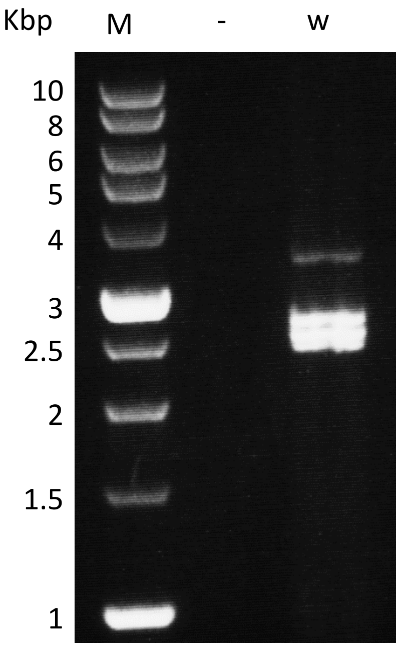

Table S3.

Parameters			Native Virus	Virus infected KU	ORF			
					1	2	3	4
Fungal phenotype	Influence at sporulation stage	Number of conidia	↓				↓	↑
		Tolerance to formate stress	↓	↓				↓
		Tolerance to hypoxic stress	↓	↓	↓			↓
		Tolerance to NO stress	↓	↓		↓		↓
		Tolerance to oxidative stress	↓	↓			↓	
		Tolerance to osmotic pressure	↓	↓			↓	↓
		Hydrophobicity	↓	↓			↓	
		Sensitivity to phagocytosis						
	Influence at germination	Swelling of conidia (6h)	↓		↑			
	Influence to mycelial growth	Radial growth (on agar plate)					↓	
		Dry weight (in fluid medium)	↓	↓	↓			
Influence to fungal virulence		Mouse lung CFU	↓	↓			↓	↑

Table S4.

Name	Sequence (5'→3')	Application
41362aF RACE A2	CTGCATCGTTGGTGCTGTTA	5'RACE
41362aF RACE P	GTGTCGTAGTTCGA	5'RACE
41362aF RACE S1	ATGTGTTGCGGTAAGTCCAC	5'RACE
41362aF RACE S2	CGACGACATCGTAGTTAACG	5'RACE
41362aR RACE A2	TGTCGATCATCGAGTCAGAG	5'RACE
41362aR RACE P	GCGTTGCGATAGAT	5'RACE
41362aR RACE S1	GAGTGTTCAATTCGCACCAG	5'RACE
41362aR RACE S2	TAGCTTTCCTGGATCTACGC	5'RACE
41362aRrealtime	TTCGTAGCTAGCACCGTTAG	5'RACE
41362bRrealtime	GCCAAGCGATGCTCAACATT	5'RACE
41362cF RACE A1	TCACGTGAAACATCGGCCTT	5'RACE
41362cF RACE A2	GGTCAATACCCAGCATGGAA	5'RACE
41362cF RACE P	TTCTCTACTCCCTG	5'RACE
41362cF RACE S1	GTGACCTTCGACCTGATGAA	5'RACE
41362cF RACE S2	AAGACCTGTGGGCATTGATG	5'RACE
41362cR RACE A1	GGGGATGGTGAGCCAATTTA	5'RACE
41362cR RACE A2	GGCAGCATTGGTCAGGTATA	5'RACE
41362cR RACE P	CAGATCAGATGGCT	5'RACE
41362cR RACE S1	TTCGGGTGGACCATGGATTA	5'RACE
41362cR RACE S2	TAAGCGATAGGGTCCGAACT	5'RACE
41362cRrealtime	CTCAACACACCCTAGCTCTT	5'RACE
41362dF RACE A2	CGACTGGGATTGATGGTGAT	5'RACE
41362dF RACE P	ACCCACCTCCATAA	5'RACE
41362dF RACE S1	ATTGGTTGGCTGCCACCAAT	5'RACE
41362dF RACE S2	GCTCTAGGCAATCGCAAGTT	5'RACE
41362dR RACE A2	TAGTCAGGCGTCGGTATCT	5'RACE
41362dR RACE P	CGAGTACGAACTGT	5'RACE
41362dR RACE S1	CTTCGCAGTCACCACTGAA	5'RACE
41362dR RACE S2	GACAGCGCGTAAGAACTGTA	5'RACE
41362dRrealtime	GAGCACCTGCAAATAATGCG	5'RACE

Table S4, continued. Primers used in this study.

Name	Sequence (5'→3')	Application
41362aFL	CCGTTCTAGTTCATCGGACA	Genome sequencing
41362aFR	ATAGCATCAAGCTCGCGGAT	Genome sequencing
41362aFR2nd	TGCGTGAACGATTCCCACTT	Genome sequencing
41362aFR3rd	CTGAACACCGAACAGTTCAC	Genome sequencing
41362aFR4th	CGTTATTCGCAAGATGAGGC	Genome sequencing
41362aRL	GATCGCTTTAATCCGTGGTC	Genome sequencing
41362aRL2nd	ATTTCGCGATCACGCTGTTG	Genome sequencing
41362aRL3rd	CGCAGCTTTGTTAAGCCTCA	Genome sequencing
41362aRR	CGGCTGCGCTACTTATATGC	Genome sequencing
41362bFL	GACGAACAAGGGATACTCGT	Genome sequencing
41362bFR	TCATTCACGTTGGGCGAAA	Genome sequencing
41362bFR2nd	TCCAGTTGAACTCAGCGATG	Genome sequencing
41362bRL	TAACACTCACGCTGCCCATA	Genome sequencing
41362bRL2nd	TCTATCCCAGACCTTGTACC	Genome sequencing
41362bRR	CGGTACTGTTCGGCTACTA	Genome sequencing
41362cFL	AAGGAGTCAGAGGCCTCTG	Genome sequencing
41362cFR	TGCTGGTCACACGAATTCAC	Genome sequencing
41362cFR2nd	TGTGGTGAAGCCTTGCGTAT	Genome sequencing
41362cRL	TTCGCCCGAAGTGTCTAGTT	Genome sequencing
41362cRL2nd	TTGAGCAGAAGCCATGCACT	Genome sequencing
41362cRR	TGTTGACTGCAATGCGTAGC	Genome sequencing
41362dFL	TAATCCTCGGGTGGTTCACA	Genome sequencing
41362dFR	CACCGGTGCTGGAAATCTAA	Genome sequencing
41362dFR2nd	TATGCTTCAGTCGACAGCGT	Genome sequencing
41362dRL	TTGCCCTGAGCTAGCTGATA	Genome sequencing
41362dRL2nd	TTTTCGGCCAGCAGATAAGC	Genome sequencing
41362dRR	CCGAAAGAAGTGAAGGGTG	Genome sequencing
41362aFR3rd	CTGAACACCGAACAGTTCAC	Probe for northern analysis
41362aRL3rd	CGCAGCTTTGTTAAGCCTCA	Probe for northern analysis
41362bFR2nd	TCCAGTTGAACTCAGCGATG	Probe for northern analysis
41362bRL2nd	TCTATCCCAGACCTTGTACC	Probe for northern analysis
41362cFR2nd	TGTGGTGAAGCCTTGCGTAT	Probe for northern analysis
41362cRL2nd	TTGAGCAGAAGCCATGCACT	Probe for northern analysis
41362dFR2nd	TATGCTTCAGTCGACAGCGT	Probe for northern analysis
41362dRL2nd	TTTTCGGCCAGCAGATAAGC	Probe for northern analysis
41362cFGeneart	TACACACTCGAGGTCATGAAGGACATCTTT	Transfomation of virus ORF genes
41362cRGeneart	CTTATCGATACCGTCCTAAGTGCCAGTACC	Transfomation of virus ORF genes
pBC1004F Hinc	GACGGTATCGATAAGCTTG	Transfomation of virus ORF genes
pBC1004R Hinc	GACCTCGAGTGTGTAGATTC	Transfomation of virus ORF genes
41362aF part2	TAACCCGGGATGAACAAGCTAACAGCACC	Transfomation of virus ORF genes
41362aR part2	AGCGGATCCCTAGTACATAGTGAAACCGC	Transfomation of virus ORF genes
41362bF part2	AAACCCGGGATGGCGAACGTACGCGTAT	Transfomation of virus ORF genes
41362bR part2	AATGGATCCCTACAAGTGCTCGCCACCTG	Transfomation of virus ORF genes
41362dF part2	AATCCCGGGTCGTCAAGTTCCAGTGACAT	Transfomation of virus ORF genes
41362dR part2	CGTGGATCCTTAAATAGCTACCCCCGTGA	Transfomation of virus ORF genes
